# Burst-Like Transcription of Mutant and Wildtype *MYH7*-Alleles as Possible Origin of Cell-to-Cell Contractile Imbalance in Hypertrophic Cardiomyopathy

**DOI:** 10.3389/fphys.2018.00359

**Published:** 2018-04-09

**Authors:** Judith Montag, Kathrin Kowalski, Mirza Makul, Pia Ernstberger, Ante Radocaj, Julia Beck, Edgar Becker, Snigdha Tripathi, Britta Keyser, Christian Mühlfeld, Kirsten Wissel, Andreas Pich, Jolanda van der Velden, Cristobal G. dos Remedios, Andreas Perrot, Antonio Francino, Francesco Navarro-López, Bernhard Brenner, Theresia Kraft

**Affiliations:** ^1^Hannover Medical School, Institute of Molecular and Cell Physiology, Hannover, Germany; ^2^Hannover Medical School, Institute of Human Genetics, Hannover, Germany; ^3^Hannover Medical School, Institute of Functional and Applied Anatomy, Hannover, Germany; ^4^Clinic for Laryngology, Rhinology and Otology, Hannover Medical School, Hannover, Germany; ^5^Hannover Medical School, Institute of Toxicology, Hannover, Germany; ^6^Department of Physiology, Institute for Cardiovascular Research, VU University, Amsterdam, Netherlands; ^7^Department of Anatomy, Bosch Institute, University of Sydney, Sydney, NSW, Australia; ^8^Cardiovascular Genetics, Experimental and Clinical Research Center, Charité-Universitätsmedizin Berlin, Berlin, Germany; ^9^Hospital Clinic/IDIBAPS, University of Barcelona, Barcelona, Spain

**Keywords:** hypertrophic cardiomyopathy, human cardiomyocytes, single-cell allelic imbalance, ventricular myosin heavy chain, myosin mutations, burst-like transcription

## Abstract

Hypertrophic Cardiomyopathy (HCM) has been related to many different mutations in more than 20 different, mostly sarcomeric proteins. While development of the HCM-phenotype is thought to be triggered by the different mutations, a common mechanism remains elusive. Studying missense-mutations in the ventricular beta-myosin heavy chain (β-MyHC, *MYH7*) we hypothesized that significant contractile heterogeneity exists among individual cardiomyocytes of HCM-patients that results from cell-to-cell variation in relative expression of mutated vs. wildtype β-MyHC. To test this hypothesis, we measured force-calcium-relationships of cardiomyocytes isolated from myocardium of heterozygous HCM-patients with either β-MyHC-mutation Arg723Gly or Arg200Val, and from healthy controls. From the myocardial samples of the HCM-patients we also obtained cryo-sections, and laser-microdissected single cardiomyocytes for quantification of mutated vs. wildtype *MYH7*-mRNA using a single cell RT-qPCR and restriction digest approach. We characterized gene transcription by visualizing active transcription sites by fluorescence *in situ* hybridization of intronic and exonic sequences of *MYH7*-pre-mRNA. For both mutations, cardiomyocytes showed large cell-to-cell variation in Ca^++^-sensitivity. Interestingly, some cardiomyocytes were essentially indistinguishable from controls what might indicate that they had no mutant β-MyHC while others had highly reduced Ca^++^-sensitivity suggesting substantial fractions of mutant β-MyHC. Single-cell *MYH7*-mRNA-quantification in cardiomyocytes of the same patients revealed high cell-to-cell variability of mutated vs. wildtype mRNA, ranging from essentially pure mutant to essentially pure wildtype *MYH7*-mRNA. We found 27% of nuclei without active transcription sites which is inconsistent with continuous gene transcription but suggests burst-like transcription of *MYH7*. Model simulations indicated that burst-like, stochastic on/off-switching of *MYH7* transcription, which is independent for mutant and wildtype alleles, could generate the observed cell-to-cell variation in the fraction of mutant vs. wildtype *MYH7*-mRNA, a similar variation in β-MyHC-protein, and highly heterogeneous Ca^++^-sensitivity of individual cardiomyocytes. In the long run, such contractile imbalance in the myocardium may well induce progressive structural distortions like cellular and myofibrillar disarray and interstitial fibrosis, as they are typically observed in HCM.

## Introduction

Hypertrophic Cardiomyopathy (HCM) is the most frequent inherited cardiac disease with a prevalence of about 1:500 (Maron et al., 1995). It is characterized by asymmetric hypertrophy of the left ventricle in the absence of other causes for hypertrophy (Maron and Maron, 2013). HCM can vary from essentially asymptomatic to highly malignant up to end-stage heart failure, or cause life threatening arrhythmias with sudden cardiac death particularly in young adults (Richardson et al., 1996). Two different presentations of HCM can be found in patients, the obstructive form (HOCM), where patients suffer from an obstruction of the left ventricular outflow tract, and the non-obstructive form (HNCM). Cardiomyocyte disarray with interstitial fibrosis and hypertrophy are hallmarks of HCM (Varnava et al., 2000). The degree of myocardial disarray correlates with risk factors for sudden cardiac death (Varnava et al., 2000), and it was suggested that myocyte disarray directly results from functional changes induced by the HCM related mutations at the sarcomeric level (Ashrafian et al., 2011).

In most familial HCM cases, heterozygous mutations in sarcomeric proteins such as the β-myosin heavy chain (β-MyHC), cardiac myosin-binding protein C (cMyBPC), cardiac troponin-T (cTnT), and cardiac troponin-I (cTnI) have been identified. Very few mutations were found in non-sarcomeric proteins (Ho et al., 2015a). About one third of the patients are heterozygous for mutations in *MYH7* (encoding β-MyHC) and *MYBPC3* (encoding cMyBPC) (Richard et al., 2003; Ho et al., 2015a).

It was proposed that the different HCM mutations lead to the HCM phenotype by enhancing contractility, and increasing calcium-sensitivity and ATPase activity of the cardiomyocytes (Ashrafian et al., 2011), while the opposite changes were thought to result in dilated cardiomyopathy (DCM) (Robinson et al., 2002; Hoskins et al., 2010; Davis et al., 2016). In studies on expressed human β-myosin with HCM-mutations also evidence for a hypercontractile state was found (Sommese et al., 2013; Bloemink et al., 2014). As mechanism for the postulated hypercontractility in HCM it was recently suggested that the mutations increase the availability of myosin heads for force production by altering the putative folded back state of the myosin heads (Kawana et al., 2017; Nag et al., 2017). Results from our group and others, however, are inconsistent with a generally enhanced contractility in HCM. Instead, contractility and calcium sensitivity can be enhanced or decreased in HCM (Venkatraman et al., 2003; Kirschner et al., 2005; Mirza et al., 2005; van Dijk et al., 2012; Kraft et al., 2013). Hence, the pathomechanism of HCM development is still unclear and a common trigger of HCM has yet to be identified.

In our work, we focused on HCM related mutations in β-MyHC which in humans is also expressed in slow twitch skeletal muscle fibers of e.g., *M. soleus*. For several missense mutations in the β-MyHC, we found reduced Ca^++^-sensitivity (Kirschner et al., 2005; Kraft et al., 2013). To our surprise, however, some muscle fibers showed a Ca^++^-sensitivity quite similar to fibers of control individuals while other fibers of the same patient had highly reduced Ca^++^-sensitivity, yielding a spectrum of different Ca^++^-sensitivities from normal to highly reduced within the same tissue sample (Kirschner et al., 2005). Based on these findings we hypothesized that significant functional heterogeneity also exists among individual cardiomyocytes of HCM-patients, and that this may result from cell-to-cell variation in expression of mutated β-MyHC. Within the myocardial cellular network, such unequal force generation among adjacent cardiomyocytes will result in distortions of cardiomyocytes and non-myocyte cells. Some of these will be overstretched or distorted by cardiomyocytes that overcontract. Such distortions could not only initiate cardiomyocyte and myofibrillar disarray but could also trigger stretch-induced signaling, e.g., TGF-β-signaling (Teekakirikul et al., 2010), that results in development of interstitial fibrosis and hypertrophy. Thus, cell-to-cell functional variance may initiate hallmarks of the HCM phenotype (Brenner et al., 2014).

In the present work, we tested our hypothesis by studying contractile properties of individual cardiomyocytes of HCM-patients' myocardium and expression of mutated *MYH7*-mRNA at the single cardiomyocyte level in the same tissue samples. We found significant functional heterogeneity in Ca^++^-sensitivity among individual cardiomyocytes of affected HCM patients, including cardiomyocytes with Ca^++^-sensitivity essentially indistinguishable from control cells. This may result from cell-to-cell variation in the fractions of expressed mutant and wildtype protein, including cardiomyocytes expressing low or almost no fraction of mutant protein. To directly test for cell-to-cell variability in expression of the mutant vs. wildtype alleles, we quantified mutant vs. wildtype transcript in individual cardiomyocytes isolated from the same tissue samples. We found substantial cell-to-cell variability ranging from essentially pure wildtype to essentially pure mutant *MYH7*-mRNA expression in the very same patients. Data from counting active transcription sites and model calculations suggest that stochastic, burst-like transcription of *MYH7*, which is independent for the mutant and the wildtype allele, could generate the large cell-to-cell variation in mutant vs. wildtype *MYH7*-mRNA and β-MyHC-protein, resulting in substantial functional heterogeneity.

## Materials and methods

For detailed methods, additional figures, and references see Supplementary Material.

### Patients and controls

This study was carried out in accordance with the recommendations of the Ethics Committee of Hannover Medical School with written informed consent from all subjects. All subjects gave written informed consent in accordance with the Declaration of Helsinki (WMA, 1997). The study on anonymized human tissue was approved by the Ethics Committee of Hannover Medical School (No. 2276-2014). Samples of left ventricular free wall and interventricular septum with β-MyHC-mutation p.R723G were from myocardium of two male HCM patients who received a heart transplant (patient II-5, family 26 and patient III-1, family 157) (Enjuto et al., 2000). A sample of the interventricular septum was obtained during myectomy from a severely affected female with Hypertrophic Obstructive Cardiomyopathy (HOCM) with the β-MyHC-mutation p.A200V. Control heart tissue from the left-ventricular free wall and interventricular septum was from non-transplanted donor hearts (*n* = 5). Cardiac tissue was flash frozen in liquid nitrogen immediately after excision.

### Single cardiomyocyte function

Cardiomyocytes were mechanically isolated and contraction parameters were measured at different Ca^++^-concentrations (pCa-values) from relaxing (pCa 9.0) to saturating Ca^++^-concentration (pCa 4.63) as previously described (Kraft et al., 2013), and (for mutation A200V) as described in Supplementary Material (Figure S1). To adjust PKA-dependent phosphorylation which has been shown previously to be higher in donor cardiac tissue compared to HCM-patient's cardiac tissue (Kraft et al., 2013), all cardiomyocytes of donors and patients were incubated with protein kinase A (PKA) prior to functional assessment. It has been shown that PKA treatment of donor cardiomyocytes induced only a small shift of the force-pCa-relation to higher calcium-concentrations, while for cardiomyocytes from patients with heart failure or HCM-patients the shift was significant (van der Velden et al., 2003; Kraft et al., 2013).

### Relative quantification of mutant vs. wildtype *MYH7*-mRNA in individual cardiomyocytes

Cryosections (thickness 5 μm) from frozen left-ventricular cardiac tissue were generated and sections of individual cardiomyocytes were isolated by laser capture microdissection after anti-cadherin staining of desmosomes (Figure S2). Individual cardiomyocytes were identified by the bright-field image clearly showing the striation pattern and were dissected when overlaying the bright-field image and the fluorescent image of intercalated discs labeled by an anti-cadherin-antibody. The cells were marked, laser-dissected, and catapulted from the tissue section into nuclease free water in the lid of a PCR-tube and lysed. Quantitative single cell RT-PCR was performed (for conditions and primers see Table S1 and Supplementary Material). The lysates were subjected to reverse transcription reaction mix and incubated on a custom-made micro-mixer for 10 min to improve uniform distribution of the low amount of mRNA molecules (Boon et al., 2011) and subsequently split into duplicates that were analyzed in parallel. The micro-mixer was also used to optimize successive cDNA synthesis (Boon et al., 2011). For relative quantification of the *MYH7* alleles in single cardiomyocytes, nested PCR was applied, followed by a reconditioning PCR to avoid heteroduplex-formation (Thompson et al., 2002). For allele specific restriction digest, R723G- or A200V-PCR-products were treated with *MboI* or *Hpy4CHI*, respectively, yielding an allele-specific band pattern. Quantification of mutant vs. wildtype *MYH7*-mRNA occurred densitometrically after testing for linearity using standard plasmids of wildtype, R723G, or A200V sequence of the PCR-amplicons as described (Tripathi et al., 2011) (Supplementary Material, Figure S3).

### Multi-aliquot control

A section of a sample from the left ventricular free wall of R723G-myocardium was lysed, diluted serially and subjected to quantitative single cell RT-PCR. The diluted lysate with a normalized IOD comparable to that of single cardiomyocytes was then divided into several aliquots for parallel quantification as described (Supplementary Material).

### *MYH7*-mRNA copy number in individual cardiomyocytes

Standard-RNA was generated by *in vitro* transcription using *MYH7*-cDNA. Single cardiomyocytes were microdissected and total *MYH7*-mRNA was determined by absolute quantification using real-time PCR and serial dilutions of the standard-RNA.

### Quantification of mutated β-myosin protein with mutation A200V in tissue samples

As described (Becker et al., 2007) (Supplementary Material; Figure S4), sarcomere-bound myosin was extracted from A200V-myocardium and digested by trypsin. Specific mutant and wildtype peptides were quantified by HPLC and mass spectrometry using corresponding synthetic stable-isotope-labeled internal standard peptides.

### Visualizing active transcription sites

Active transcription sites were visualized by fluorescence *in situ* hybridization (FISH) using sets of 48 20-mer oligonucleotides (Stellaris®-probes; LGC Biosearch Technologies, Petaluma, CA, USA). One set was designed to hybridize with intronic sequences of *MYH7*-pre-mRNA and each oligonucleotide was labeled with one Cy5-like fluorophore (Quasar 670, LGC Biosearch Technologies). The other set was designed to hybridize with exonic sequences of *MYH7*-mRNA and labeled with a Cy3-like fluorophore (Quasar 570; LGC Biosearch Technologies). Both probe sets were custom made (Stellaris® Probe Designer). Following hybridization, active transcription sites were taken as bright spots inside nuclei of cardiomyocytes showing both fluorescence signals. Further details are described in Supplementary Material.

### Modeling of independent, burst-like transcription of mutant, and wildtype *MYH7*-alleles

Model calculations were based on the concept of stochastic, burst-like transcription (Raj et al., 2006) including independent transcription and translation of mutant and wildtype *MYH7*-alleles to account for our experimentally observed mutant vs. wildtype transcript levels and function of individual cardiomyocytes from the heterozygous R723G-patient II-5. Modeling included the stochastic opening and closing of the transcription sites, synthesis of pre-mRNA, splicing to mRNA, degradation of mRNA, and synthesis and degradation of protein, each for mutant and wildtype, respectively. The only adjustable parameters were the rate constants for activation/inactivation of transcription of the two alleles and the splicing rate constant; all other rate constants were taken from the literature. From the measured fraction of mutated β-MyHC-protein in R723G-patient myocardium and the mean pCa_50_-values of controls and R723G-patient cardiomyocytes we could also simulate a distribution of pCa_50_-values. For details on modeling constraints, additional results and references see Supplementary Material.

### Statistical analysis

Data are presented as mean ± SD or ± 1.96 SD (range in which 95% of data points are expected; see Supplementary Material). To assure normal distribution (Shapiro-Wilk test) of normalized data, logit transformation was performed for statistics (Ashton, W. D., 1972). Student's *t*-test was used to determine significance levels. Equality of variances was examined by *F*-test. For *p* < 0.05 significance was assumed.

## Results

### Large cell-to-cell functional heterogeneity among individual cardiomyocytes from HCM patients

#### Force-pCa relations

**Figure 1:**
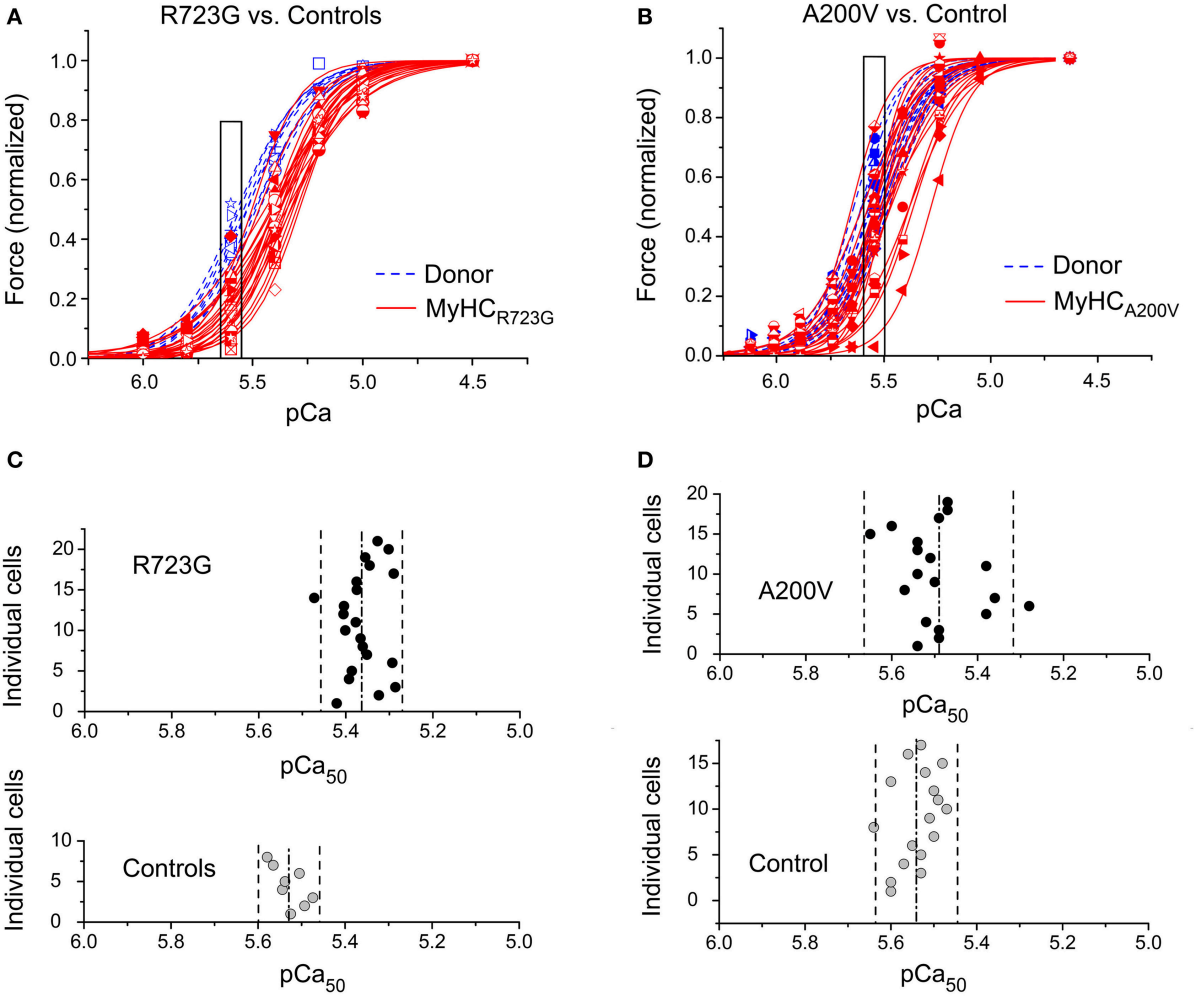
Force generation by individual cardiomyocytes. Forces of individual left ventricular cardiomyocytes at different calcium-concentrations (force-pCa-relations), normalized to maximum force, with Hill-functions fitted to data points. **(A)** β-MyHC-mutation R723G (*n* = 22) vs. controls (*n* = 8) and **(B)** β-MyHC-mutation A200V (*n* = 19) vs. controls (*n* = 17). Mutated cardiomyocytes, *red symbols and red solid lines*; controls, *blue symbols and blue dashed lines*. Different symbols represent different individual cells. The mean force-pCa-relationships are shown in Supplementary Material, Figure S5. **(C)** pCa_50_ from individually fitted Hill-equations for R723G-cardiomyocytes and **(D)** for A200V-cardiomyocytes vs. controls. Dash-dot lines represent mean pCa_50_-values (cf. Table 1); dashed lines delineate range in which 95% of all data points (mean ± 1.96 SD) are expected. pCa_50_-values of all groups show normal distribution (Shapiro-Wilk test). SD of pCa_50_-values of mutated cardiomyocytes larger than that of controls (for A200V-cardiomyocytes statistically significant, *p* = 0.02, F-test).

Cell-to-cell functional heterogeneity among individual cardiomyocytes of HCM patients was investigated by recording force-pCa relations of cardiomyocytes isolated from myocardial samples with β-MyHC-mutations R723G (Enjuto et al., 2000) and A200V, respectively, and of healthy controls for comparison (Figure 1). Force data of each cardiomyocyte were normalized to the maximum force at saturating Ca^++^-concentration (pCa 4.5). Interestingly, force-pCa relations of some cardiomyocytes with mutation R723G were similar to that of donor cells, while for others a clear shift to higher Ca^++^-concentrations was observed (Figure 1A). The mean shift to reduced Ca^++^sensitivity with mutation R723G (Figure S5A) was similar to the change we had previously found in fibers of *M. soleus* muscle and in myocardium of this and other patients with mutation R723G (Kirschner et al., 2005; Kraft et al., 2013). For cardiomyocytes with mutation A200V we found a similar pattern with some force-pCa relations comparable to donor cells and others with markedly reduced Ca^++^-sensitivity (Figure 1B). On average, the force-pCa curve for A200V was slightly shifted to higher Ca^++^-concentrations, however, due to the larger cell-to-cell variability the shift is not statistically significant (Figure S5B).

For both mutations, the individual force-pCa relations reveal a larger cell-to-cell variability in the position of the force-pCa relation along the abscissa, i.e., a larger variability in Ca^++^-sensitivity than in control cardiomyocytes (Figures 1A,B). This is particularly prominent for mutation A200V for which force-pCa relations vary from the range of the control myocytes up to positions shifted by about 0.25 pCa units to the right (red solid lines in Figure 1B). Thus, for both mutations some cardiomyocytes have a Ca^++^-sensitivity indistinguishable from that of control cells while other cells have a substantially lower Ca^++^-sensitivity. This is further illustrated by the pCa_50_-values of the individual cardiomyocytes obtained from fitting the Hill equation to the force data of the individual cells. In Figures 1C,D the pCa_50_-values of the individual cardiomyocytes are shown together with the related mean value and the range in which 95% of data points are expected (mean ± 1.96 SD). Overall, the pCa_50_-values of individual R723G and A200V cardiomyocytes are significantly lower than those of the related control cells, respectively, and the variances of the pCa_50_-values are larger than those for controls. For the A200V cells, the variance is significantly larger than for controls (*p* = 0.02; *F*-test).

**Table 1:** Average pCa_50_ values of cardiomyocytes from controls and from HCM patients with β-MyHC mutation R723G and A200V, respectively.

**pCa**_**50**_	
**R723G**	**Controls**
5.38 ± 0.05^***^	5.53 ± 0.04
*n* = 22	*n* = 8
**A200V**	**Controls**
5.49 ± 0.09^*^	5.54 ± 0.05
*n* = 19	*n* = 17

*** p < 0.001;

* *p = 0.044; n indicates number of cardiomyocytes*.

#### Forces at partial activation

Functional consequences of heterogeneities in the force-pCa relations of individual cardiomyocytes are illustrated when the forces generated by individual R723G- and control-cardiomyocytes are compared at partial activation level (pCa 5.55–5.6; boxed areas in Figures 1A,B). The pCa-values 5.5–5.6 represent physiological intracellular Ca^++^-concentrations in a twitch (Fabiato, 1981).

For both mutations, the mean relative forces at pCa 5.55–5.6 are significantly lower compared to controls (p_R723G_ < 0.001, p_A200V_ = 0.025; *t*-test). For cardiomyocytes of both patients, variances of forces at partial activation are significantly higher than that of respective controls (R723G *p* = 0.003; A200V *p* = 0.002; *F*-test). In fact, at pCa 5.55–5.6 forces generated by the “weakest” and “strongest” HCM cardiomyocytes were 10-fold (R723G) or even 20-fold (A200V) different (boxed areas in Figures 1A,B). In contrast, at the same partial activation, forces of weakest and strongest control cardiomyocytes differed at most 1.5-fold (Figures 1A,B). Since normalized forces are restricted to values between 0 and 1, statistical analysis was done after logit transformation (Ashton, W. D., 1972). For details see Supplementary Material. The variance of the control cardiomyocytes reflects experimental error, while the much larger functional variance among individual cardiomyocytes of both HCM patients very likely results from an additional, large intrinsic cell-to-cell heterogeneity of their calcium-sensitivity.

This large intrinsic heterogeneity among individual cardiomyocytes of HCM patients, where some cells were very similar to controls and others had very much reduced Ca^++^-sensitivity, raised the question whether this reflects variation from small to rather high fractions of mutated vs. wildtype protein in these cardiomyocytes, respectively.

### Highly heterogeneous fraction of mutant *MYH7*-mRNA from cell-to-cell

Since protein quantification of mutant vs. wildtype β-MyHC with missense mutations in individual cardiomyocytes is beyond the sensitivity of our mass-spectrometry approach (Becker et al., 2007; Tripathi et al., 2011), we quantified relative expression of mutant and wildtype *MYH7*-alleles in individual cardiomyocytes at the mRNA level. We expect this to reflect expression at the protein level, since in our previous work on several β-MyHC-mutations including several patients with mutation R723G we always found a nearly 1:1 relation between fraction of mutant protein and mutant mRNA at the tissue level (Figure S7B) (Tripathi et al., 2011; Montag et al., 2017). Such a 1:1 relation between *MYH7*-mRNA and β-MyHC-protein was also found here and previously for mutation A200V (see below and Supplementary Material, Figure S7).

As the myocardial tissue samples had been flash-frozen immediately after surgery, we could not enzymatically isolate intact individual cardiomyocytes to determine the fraction of mutant *MYH7*-mRNA. Instead, individual cardiomyocytes were isolated from cardiac tissue sections by laser capture microdissection (Figure S2). Quantification of each cell was performed in duplicates. A specific micro-mixing method was used to optimize cDNA synthesis (Boon et al., 2011). To quantify the relative abundance of mutant vs. wildtype *MYH7*-mRNA in cryosections of individual cardiomyocytes, we adapted the RT-PCR/restriction digest approach that we had previously used for quantification of *MYH7*-mRNA in tissue samples (Tripathi et al., 2011).

**Figure 2:**
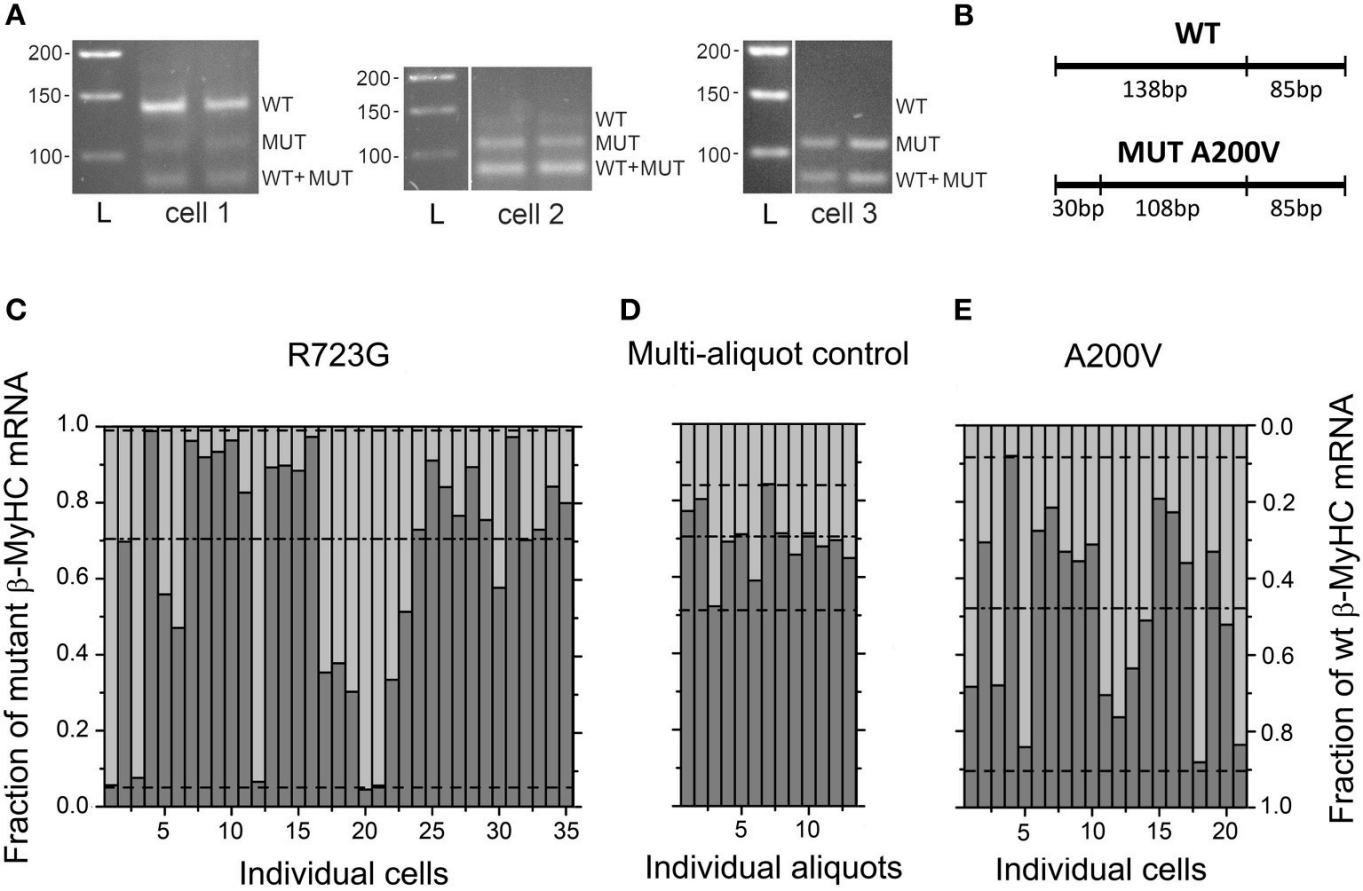
Fraction of mutant *MYH7*-mRNA and fraction of wildtype *MYH7*-mRNA in individual cardiomyocytes microdissected from cryosections of myocardium. **(A)** Gels of restriction digest products of 3 individual cardiomyocytes microdissected from cryo-sections of A200V-myocardium. Lysate of each cell was divided in 2 equal aliquots and analyzed in parallel; *L*, DNA standard ladder. Note the marked differences in band pattern among different individual cardiomyocytes (cell 1-3) while band patterns of the two aliquots of each cell are rather similar. **(B)** *Schematic* of *HpyCH4III*-restriction sites: 138 bp band, from wildtype *MYH7*-mRNA; 108 bp band, from mutant *MYH7*-mRNA; 85 bp band, from mutant and wildtype mRNA; 30 bp band, from mutant *MYH7*-mRNA but outside range of gels. **(C)** Fraction of R723G-mRNA (dark gray bars; y-axis on the left of panel) and of wildtype-mRNA (light gray bars; y-axis on the right of panel) in 35 cardiomyocytes from left ventricular free wall of R723G-myocardium. *MYH7*-mRNA varies from almost pure wildtype to pure mutant. Statistics on mean values of the two aliquots after logit transformation (see text). **(D)** 13 Aliquots from cDNA of R723G-myocardium (left ventricular free wall) analyzed in parallel to test for experimental scatter. Average *MYH7*-mRNA-fraction is very similar to average of individual cardiomyocytes. **(E)** Fraction of A200V-mRNA (dark gray bars) and of wildtype-mRNA (light gray bars) in 21 cardiomyocytes of A200V myectomy-sample. Fraction varies from ≈10 to 90%. Statistics as in **(C)**. Dash-dot line and dashed lines, means ± 1.96 SD. Normal distribution of individual cells and multi-aliquot data (Shapiro-Wilk test). Cell-to-cell variance in fraction of mutated *MYH7*-mRNA for individual cardiomyocytes significantly larger than variance of multi-aliquot control (experimental error); for R723G, *p* < 0.0001; for A200V, *p* < 0.001 (*F*-test).

Three representative restriction analyses of individual A200V-cardiomyocytes in Figure 2A show that the fraction of mutant β-cardiac mRNA varies among individual cardiomyocytes from quite low mutant (cell 1) to almost pure mutant *MYH7*-mRNA (cell 3). Figure 2B schematically shows the fragments generated by the A200V-specific restriction digest. Note, the highly similar band pattern of the two aliquots of each individual cardiomyocyte indicates that the large cell-to-cell variability in the abundance of mutant *MYH7*-mRNA is not due to experimental error. For a similar sample gel with R723G-cardiomyocytes see Figure S6 and previous work for the other patient (Kraft et al., 2016).

Analysis of in total 35 individual R723G-cardiomyocytes showed that the fraction of mutant *MYH7*-mRNA varies from essentially pure mutant to almost pure wildtype *MYH7*-mRNA (Figure 2C). This was confirmed for another patient with the same mutation from another family (Figure S6). To obtain an estimate for the experimental error in our mRNA quantification procedure, we generated a “tissue averaged” cDNA sample that could be divided into multiple aliquots for parallel analysis following the same procedure as for the single cardiomyocytes. A whole cryosection of R723G-tissue was lysed and cDNA was synthesized. This cDNA sample was diluted and then divided into aliquots such that each aliquot contained a similar amount of cDNA as we obtained from individual microdissected cardiomyocytes (for details see Supplementary Material). Thus, these aliquots yielded band intensities of the restriction digest that were similar to those observed with microdissected individual cardiomyocytes. The fractions of R723G-*MYH7*-mRNA in each of the 13 aliquots ranged only between 0.5 and 0.85 (Figure 2D).

Since the fraction of mutant *MYH7*-mRNA (F_mut_mRNA_) is restricted to the range between 0 and 1, for statistical analysis F_mut_mRNA_ was transformed into logits = ln((F_mut_mRNA_)/(1-F_mut_mRNA_)). For the 35 microdissected cardiomyocytes, F_mut_mRNA_ is the average of the fraction observed in the two aliquots. Data of both groups, the 35 individual microdissected R723G-cardiomyocytes and the 13 control samples, showed normal distribution after logit transformation (Shapiro-Wilk test). Importantly, the variance in the 13 aliquots of the large cDNA sample, representing the experimental error, is significantly smaller than the variance in the fraction of R723G-*MYH7*-mRNA among the 35 individual microdissected cardiomyocytes (Figures 2C,D; *p* < 0.0001, *F*-test). In contrast, the mean fraction of mutated *MYH7*-mRNA of the aliquots with 0.69 was comparable to the mean of the individual cardiomyocytes with 0.70.

Cell-to-cell variance in the fraction of mutant A200V-*MYH7*-mRNA is shown in Figure 2E. Isolation of individual cardiomyocytes and quantitative single cell RT-PCR were performed as described for R723G, with primers and endonuclease adapted for A200V. Quantitative analysis of 21 microdissected cardiomyocytes again revealed a much larger cell-to-cell variation in the fraction of mutant mRNA (Figure 2E) than seen in the 13-aliquot control. Together, R723G- and A200V-data reveal a large variability among individual cardiomyocytes in the expression of the mutated allele relative to the wildtype allele, i.e., a large cell-to-cell variance of mutant transcript levels in the myocardium of the HCM patients. The range of variability for mutant A200V is somewhat smaller than for mutant R723G (Figure 2C). We did not see very high fractions of mutated A200V-mRNA which could be due to the fact that the mean fraction of mutated mRNA in A200V cells and tissue is lower than for R723G (dash-dot lines in Figures 2C,E, Figure S7).

Analysis of the average fraction of mutant mRNA in all analyzed cardiomyocytes together yielded a fraction of 0.70 for the R723G-patient (dashed-dot-line in Figure 2C, Figure S7A). This is very similar to the value determined earlier in whole tissue samples of several patients with R723G at the mRNA and protein level (four *M. soleus* and one other cardiac sample) and reflects the allelic imbalance at the tissue level described previously (Tripathi et al., 2011) (Figure S7). The higher abundance of mutant *MYH7*-mRNA compared to wildtype *MYH7*-mRNA may be the result of higher stability (longer life-time) of the mutant *MYH7*-mRNA (Tripathi et al., 2011).

The average fraction of A200V-mRNA of all analyzed cardiomyocytes was 0.53 (Figure 2E, Figure S7A), which is very close to the mean value determined in three whole cryosections of A200V-myocardium of 0.47 ± 0.05 (Figure S7A) and from larger tissue samples of the same myocardium of 0.48 ± 0.02 (Montag et al., 2017). We also determined the fraction of mutated protein in the myectomy sample with mutation A200V. The analysis was performed by mass spectrometry as described previously (Becker et al., 2007) (for peptides and enzyme see Supplementary Material) and yielded a fraction of 0.54 of A200V-β-myosin (Figure S7), which is similar to the previously determined value of 0.49 ± 0.01 (Montag et al., 2017). Thus, also for mutation A200V the mean fraction of mutated *MYH7*-mRNA and β-MyHC-protein in myocardial tissue samples is essentially the same, as found for several other β-MyHC-mutations before (Tripathi et al., 2011).

### Independent, stochastic on-off switching of transcription of mutant, and wildtype *MYH7*-alleles as possible cause for cell-to-cell mRNA and functional heterogeneity

Large variability in mRNA expression with functional heterogeneity among genetically identical cells was previously proposed to be the result of burst-like, stochastic transcription (Raj et al., 2006).

**Figure 3:**
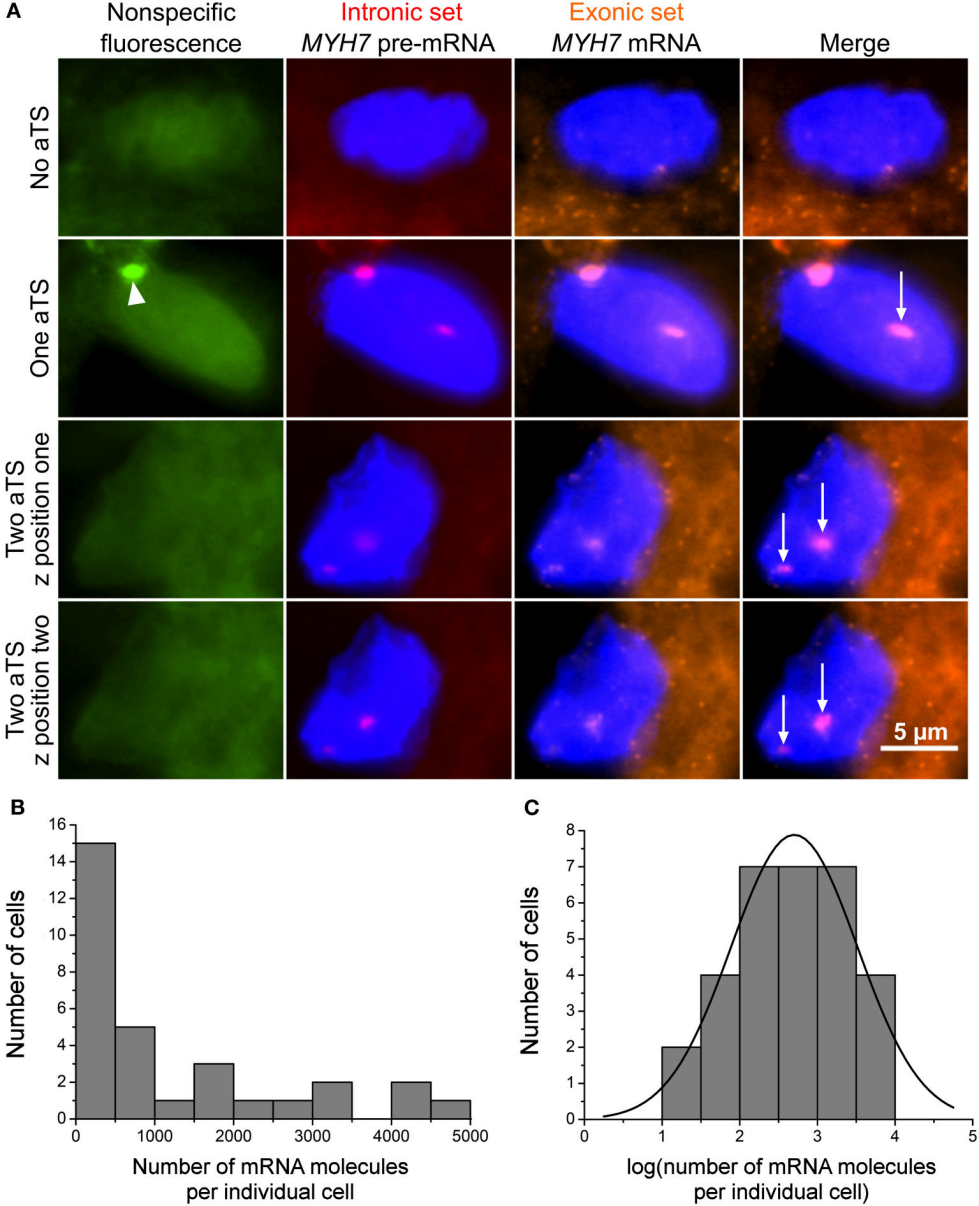
Cardiomyocyte nuclei with 0, 1, and 2 active transcription sites, and distribution of total *MYH7*-mRNA copy number in 31 individual cardiomyocytes. **(A)** Fluorescence *in situ* hybridization (FISH) to visualize active transcription sites (aTS) in 16 μm thick sections of R723G cardiac tissue samples. Arrows pointing at active transcription sites with co-localization of intronic pre-mRNA signal (red) and exonic mRNA signal (orange). Orange spots in the cytoplasm indicate individual *MYH7*-mRNA molecules (exonic sequence only), identifying cells as cardiomyocytes. Shown is an example of a nucleus without aTS (upper row), a nucleus with one aTS (2nd row; arrow head indicates non-specific fluorescent spot visible in all channels), and a nucleus with two aTS (3rd and 4th row). Note that the two aTS are located at somewhat different z-levels, as indicated by the two slices of the z-stack shown here. In this analysis, we included only nuclei that were fully embedded in the tissue sections. DAPI (nuclear stain) in blue; test for non-specific fluorescence signals in green (clearly visible in leftmost images only, except bright spot of non-specific fluorescence labeled by arrow head in 2nd row). Scale bar, 5 μm. **(B,C)** Total *MYH7*-mRNA copy numbers of 31 cardiomyocytes microdissected from sections (thickness 5 μm) of R723G cardiac tissue were quantified. Note that the distribution on a linear scale in **(B)** is not a normal distribution. Distribution of log_10_ of total *MYH7*-mRNA copy number in **(C)** can well be fit by a normal distribution (solid line), which is characteristic for burst-like transcription (Raj and van Oudenaarden, 2009).

To test if this proposed mechanism of transcription also applies to *MYH7* and transcription of *MYH7* is indeed discontinuous, interrupted and burst-like, we visualized active transcription sites in the nuclei of cardiomyocytes. For this we used fluorescence *in situ* hybridization of cryosections of R723G-cardiac tissue samples with two fluorescently labeled 20-mer oligonucleotide probe sets. In active transcription sites within nuclei pre-mRNA contains both intronic and exonic sequences. One of the two probe sets was designed to hybridize with 48 intronic sequences of the *MYH7*-pre-mRNA. The other probe set targeted 48 exonic sequences thus labeling both *MYH7*-pre-mRNA as well as *MYH7*-mRNA. Active transcription sites were identified as bright spots inside the nuclei of cardiomyocytes showing co-localization of both probe sets (Figure 3A). Cardiomyocytes were identified by the abundant presence of cytoplasmic spots of the exonic probe set indicating presence of *MYH7*-mRNA molecules. We counted active transcription sites in 122 nuclei of cardiomyocytes in R723G 16 μm-cryosections (13 cryosections; two R723G cardiac tissue samples) by analyzing 3D-stacks recorded by epifluorescence microscopy (Figure 3A) (Bahar Halpern et al., 2015). Of these 122 nuclei 27% had no active transcription sites but cytoplasmic *MYH7*-mRNA. Only nuclei fully embedded in the tissue sections were included in the analysis. In a control sample (5 cryosections) of a non-transplanted heart we found 32% of 240 nuclei in cardiomyocytes without active transcription sites. Absence of active transcription sites is inconsistent with continuous transcription of the two *MYH7*-alleles but is expected for discontinuous, stochastic, burst-like transcription.

This finding is supported by the distribution of the *MYH7*-mRNA copy number in individual cells (Figure 3B,C). We quantified the total copy number of *MYH7*-mRNA in 31 individual cardiomyocytes microdissected from cryosections of R723G cardiac tissue, thereby finding copy numbers per cardiomyocyte-slice from <10 up to 4.660. Figures 3B,C show this distribution on a linear and log scale. The distribution on the log scale is consistent with a normal distribution (Shapiro-Wilk test); the solid line represents a Gaussian fit to the decimal logarithm of the *MYH7*-mRNA copy number per cell. This log-normal distribution suggests a stochastic, burst-like transcription mechanism (Raj et al., 2006; Raj and van Oudenaarden, 2008; Bahar Halpern et al., 2015), while continuous transcription of the alleles is expected to generate a Poisson-distribution on the linear scale which is not consistent with our data (Figure 3C). Since here slices were 5 μm thick, not the full cell volume was included in the analysis. From the average diameter of a cardiomyocyte (16.9 ± 1.3 μm; Olivetti et al., 1996) the mRNA-copy number per cell should be about 3-times larger than determined in the slices.

### Numerical model simulation of cell-to-cell variation of mutant mRNA, mutant protein, and functional variability based on stochastic on/off switching of transcription sites

Next we asked whether our experimental observations could be accounted for by stochastic, burst-like transcription of the *MYH7*-alleles, which is independent for the mutant and wildtype allele. These experimental observations include the large cell-to-cell variation in fraction of mutant vs. wildtype *MYH7*-mRNA (Figure 2), the cell-to-cell functional imbalance (Figure 1), and the 27% of R723G-cardiomyocyte nuclei having no active transcription sites (Figure 3). To address the above question we set up a numerical simulation for mutation R723G in which the mutant and wildtype *MYH7*-alleles were switched on and off stochastically and independently of each other. In addition, production of pre-mRNA, splicing to mRNA, and mRNA decay were also included in the model as was translation to and decay of β-MyHC protein. For details of the simulations see Supplementary Material. The rate constants for synthesis of pre-mRNA, for degradation of mRNA, as well as for synthesis and degradation of protein were all taken from the literature, and are listed together with the respective references in Table S3. The only parameters we could use to fit the response of our simulations to the experimentally observed data were the rate constants for the stochastic switching on and off of transcription of the two alleles, and the rate constant of splicing of pre-mRNA to mRNA that were not available from the literature. These three parameters were adjusted to produce the best fit to all our above stated experimental findings.

Since a substantial fraction of cardiomyocytes can be polyploid, particularly in hypertrophied myocardium (Brodsky et al., 1994), we determined the ploidy of the R723G cardiomyocytes and included the observed distribution of di-, tetra-, octo- 16-, and 32-ploid nuclei in our simulation (for details see Supplementary Material).

A sample time course obtained from the model simulation of the transcription bursts, the mutant and wildtype *MYH7*-pre-mRNA, the mutant and wildtype *MYH7*-mRNA, the fraction of mutant *MYH7*-mRNA, and the fraction of mutant protein are shown for mutation R723G in Figure S8 for a diploid and tetraploid cardiomyocyte. To account for a tissue-wide average fraction of around 0.67 for both R723G *MYH7*-mRNA and R723G β-MyHC (Tripathi et al., 2011; Figure S7), we adjusted the mRNA decay rate of the R723G *MYH7*-mRNA to half of that of wildtype *MYH7*-mRNA.

**Figure 4:**
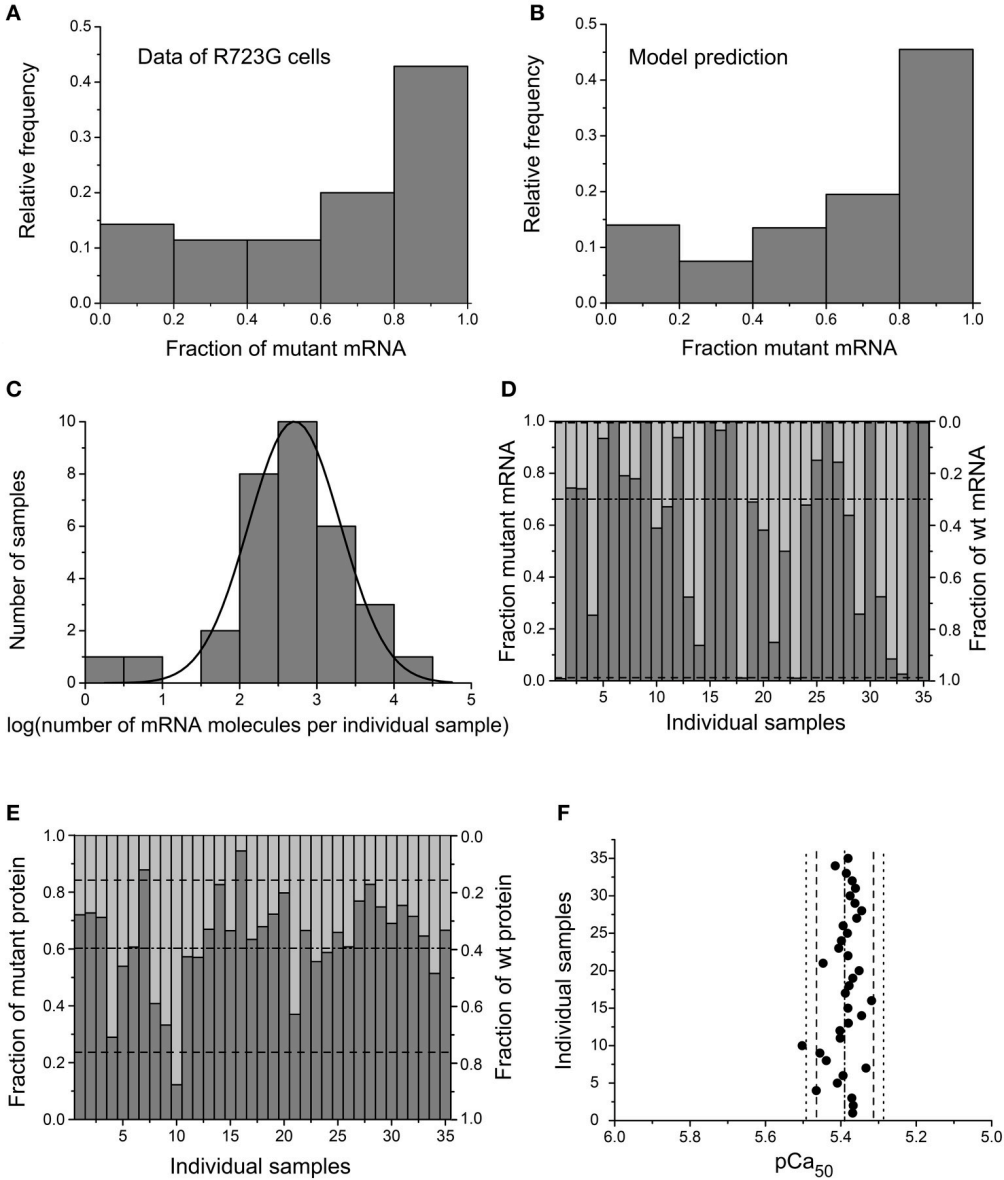
Predictions of independent, stochastic, burst-like transcription of mutant and wildtype *MYH7*-alleles. Histograms (frequency distributions) of **(A)** observed fraction of mutant *MYH7*-mRNA in the 35 microdissected R723G cardiomyocytes (cf. Figure 2C) and of **(B)** fraction of mutant *MYH7*-mRNA at 35 randomly picked points of a very long model simulation of transcription with independent, stochastic on/off switching of mutant and wildtype alleles. **(C)** Log-normal distribution of total copy number of *MYH7*-mRNA obtained from the model simulation at the 35 points, simulating cardiomyocytes microdissected from 5 μm thick cryo-sections. Simulated numbers were divided by 3 to account for 5 μm thickness of patient's tissue sections that represents only about 1/3 of full cardiomyocyte volume. Distribution slightly narrower than for experimental quantification (cf. Figure 3C). **(D)** Predicted fraction of mutant and wildtype *MYH7*-mRNA (cf. Figure 2C) and **(E)** predicted fraction of mutant and wildtype β-MyHC (protein) at the 35 randomly picked points; dash-dot line and dashed lines, means ±1.96 SD. In **(D**,**E)** dark gray bars indicate fraction of mutant *MYH7*-mRNA **(D)** and mutant protein **(E)** (y-axis on the left), and light gray areas indicate fraction of wildtype *MYH7*-mRNA **(D)** and wildtype protein **(E)** (y-axis on the right). **(F)** Predicted distribution of pCa_50_-values (filled circles) as expected from an experimentally determined mean fraction of mutant β-MyHC-protein of 0.67 (Tripathi et al., 2011) that shifts pCa_50_ by 0.15 pCa-units to the right (Figure S5A) without taking experimental error into account (dash-dot lines, mean; dashed lines, ±1.96 SD). Dotted lines, 95% range when taking experimental error into account (for details see Supplementary Material). Note the very similar 95% range as in Figure 1C for R723G cardiomyocytes.

To be able to directly compare the results of 35 individual cardiomyocytes flash frozen and microdissected from myocardial tissue to the outcome of the simulation (fractions of mutant *MYH7*-mRNA and mutant β-MyHC protein), we randomly picked 35 points of a very long simulation run (see Supplementary Material). A large separation between the randomly picked points was used to assure 35 uncorrelated, independent points, just like in individual cardiomyocytes at the time of freezing of the myocardial tissue. Comparing the frequency distribution of the observed fractions of mutant mRNA in our experimentally analyzed cardiomyocytes (Figure 4A) with the outcome of our model (Figure 4B) shows that the distributions are very similar. Likewise, the values for the total number of *MYH7*-mRNA molecules and their frequency distribution obtained from the simulation at the randomly picked 35 points (Figure 4C) are similar to the experimental data (cf. Figure 3C). Thereby we have to consider that in 5 μm cell slices the mRNA copy number is only about 1/3 of that in whole cells with a mean diameter about 16–17 μm (Olivetti et al., 1996). Also, the predicted fractions of mutant mRNA from the 35 randomly picked points (Figure 4D) match the experimentally determined values for individual cardiomyocytes (cf. Figure 2C).

We next aimed to predict the fraction of mutant β-MyHC-protein per individual cell and its effect on β-MyHC function. Intriguingly, our model predicts that at the 35 randomly picked points the value for the fraction of mutant β-MyHC-protein ranges from 0.25 to 0.87, thus suggesting a marked variation from cell to cell (Figure 4E). This is in accordance with our hypothesis that different fractions of mutant β-MyHC may cause the observed functional differences among individual cardiomyocytes. We finally used the predicted fractions of mutant protein to calculate the expected shift in pCa_50_ for each cell (Figure 4F, for calculation see Supplementary Material). Note, the mean value of the predicted pCa_50_ is essentially identical with the experimentally determined one (Figure 1C). However, the range in which 95% of all data points are expected is somewhat narrower in the model than seen experimentally (dashed lines in Figure 4F vs. dashed lines in Figure 1C). This is because in the modeling only intrinsic variance of the pCa_50_ arising from different expression of mutant β-MyHC-protein among individual R723G-sample cardiomyocytes is considered. If experimental error (represented by the variance in pCa_50_ of control cardiomyocytes, cf. Figure 1C, lower panel) is also taken into account, this results in a 95% range that is very similar to the one in the experimental data (dotted lines in Figure 4F and dashed lines in Figure 1C).

Finally, with the best fit to all our experimental results a fraction of 24% nuclei without active transcription sites was predicted, which is close to the 27% seen experimentally. This fraction is strongly dependent on the two rate constants for the on/off-switching of the mutant and wildtype *MYH7*-alleles, and is just slightly modulated by the splicing rate constant from pre-mRNA to mRNA. The on-times of mutant and wildtype *MYH7*-alleles generate “spikes” of pre-mRNA. These are substantially shorter than the spikes of mRNA which are longer due to the slower mRNA decay rate. Similarly, lifetime of the β-MyHC protein is substantially longer than of *MYH7*-mRNA, resulting in smaller fluctuations of the fraction of mutant β-MyHC protein than of mutant *MYH7*-mRNA (Figure S8).

## Discussion

In this study, we tested the hypothesis that a functional heterogeneity exists among individual cardiomyocytes in myocardium of HCM patients that results from cell-to-cell variation in the fraction of mutant β-MyHC. Functional heterogeneity with imbalance in force generation during twitches among neighboring cardiomyocytes may well contribute to development of disarray, hypertrophy and fibrosis in HCM in the long run.

The main findings of our present study are (i) a quite substantial heterogeneity in Ca^++^-sensitivity among individual cardiomyocytes with two different HCM related β-MyHC-mutations, mutation R723G and mutation A200V, respectively (Figure 1), (ii) large cell-to-cell variation in the fraction of mutant *MYH7*-mRNA among individual cardiomyocytes isolated from the same tissue samples (Figure 2), and (iii) absence of active transcription sites in 27% of nuclei in R723G cardiomyocytes indicating discontinuous, stochastic, burst-like transcription of the two *MYH7*-alleles (Figure 3). (iv) To test for an underlying mechanism that may provide a link between the experimental findings, numerical simulations were set up. They suggest that stochastic on/off switching of transcription of *MYH7*, which is independent for the mutant and the wildtype allele, can result in the observed cell-to-cell variation in mutant *MYH7*-mRNA and protein. Since the mutation reduces calcium-sensitivity, the variation in mutant myosin may also cause highly heterogeneous Ca^++^-sensitivity among individual cardiomyocytes (Figure 4). Thus, independent, stochastic on/off switching of transcription could reproduce the experimentally observed significant functional imbalance among individual cardiomyocytes isolated from the same piece of myocardial tissue as well as the observed fraction of nuclei without active transcription sites.

Our data and modeling suggest that the observed cell-to-cell variability in the fraction of mutated and wildtype *MYH7*-mRNA corresponds to a similar variability in the fraction of mutated and wildtype β-MyHC at the protein level, although the extent of this variability is smoothened by the lifetime of the protein (cf. Figure S8). Thus, since the observed fraction of *MYH7*-mRNA ranged from near zero (0.05) to essentially pure (1.0) mutant mRNA, the predicted protein fractions will presumably range from about 0.25 to 0.90, which represents a remarkable difference between the individual cells. Although protein quantification was not possible for individual cardiomyocytes, already the functional data corroborate this prediction. The calcium sensitivity of some cardiomyocytes was indistinguishable from controls (Figure 1). This suggests that these cardiomyocytes had little mutant myosin but mostly wildtype myosin in their sarcomeres at the time of freezing of the sample. On the other hand, cardiomyocytes with the largest shift of the force-pCa-curve most likely contained quite a large fraction of mutant myosin.

The correspondence of the mean fraction of mutated protein to the mean fraction of mutated *MYH7*-mRNA is further supported by our previous work on tissues of several HCM patients with β-MyHC-mutations including cardiac samples with mutation R723G (Tripathi et al., 2011), and by protein quantification of tissue samples with mutation A200V (Figure S7) (Montag et al., 2017). In all cases a close correlation between the mean fractions of mutant *MYH7*-mRNA and mutant protein was found.

Importantly, the populations of individual cardiomyocytes from which we determined the fraction of mutant *MYH7*-mRNA are representative for the cardiomyocyte populations in larger tissue samples. This is evident from the average fractions of mutant *MYH7*-mRNA of the individual cardiomyocytes, 0.70 for R723G mutation and 0.53 for A200V cardiomyocytes (Figures 2C,E), which are very close to the fractions determined in larger tissue samples, 0.70 for R723G and 0.47 for A200V (Figure S7) (Montag et al., 2017).

### Possible mechanism for cell-to-cell variation in mutant *MYH7*-mRNA and β-MyHC-protein

Continuous expression of both alleles could only account for the observed large variation in the fraction of mutant *MYH7*-mRNA among individual cardiomyocytes if only a low copy number (<50) of the *MYH7*-mRNA was generated per cardiomyocyte (Raj and van Oudenaarden, 2009). Also, continuous expression is expected to generate a Poisson distribution of the mRNA copy number per cell (Raj and van Oudenaarden, 2009). Both points, however, are inconsistent with our data. Absolute quantification revealed a log-normal distribution with a mean of 416 *MYH7*-mRNA molecules per cell in the 5 μm tissue sections (Figure 3), yielding an expected median of about 1,200 mRNA molecules per cell.

Another possibility to account for the large cell-to-cell variation in mutant/wildtype *MYH7*-mRNA is random, monoallelic expression of the *MYH7*-gene. In fact, for fractions of mutant *MYH7*-mRNA ≥ 0.9 and ≤ 0.1 we cannot rule out that the small amount of wildtype or mutant mRNA, respectively, results from cross-contamination in cryosectioning and microdissection. That a large number of cardiomyocytes, however, have fractions of mutant *MYH7*-mRNA between 0.1 and 0.9 makes monoallelic expression rather unlikely. In addition, we also determined cells with more than one active transcription site, which seems incompatible with monoallelic expression. The functional data also argue against monoallelic expression as we do not see two clearly separate groups of force-pCa relations, one like controls, the other clustering around a lower pCa_50_ value. We rather see a continuum of curves (Figure 1).

Therefore, the mechanism responsible for the large variance in the fraction of mutant *MYH7*-mRNA should account for both, cardiomyocytes with mixed mutant and wild-type *MYH7*-mRNA expression, and for essentially monoallelic expression. Using model simulations we show that stochastic, burst-like transcription of the mutant, and wildtype *MYH7* alleles, due to stochastic opening and closing of chromatin (Janicki et al., 2004), can account for our experimental data. Burst-like transcription has been described in eukaryotic (Chubb et al., 2006) and mammalian cells (Raj et al., 2006), as well as in intact mammalian tissue (Raj et al., 2006; Bahar Halpern et al., 2015). This mechanism has been proposed as the basis for large cell-to-cell variation in the number of specific protein molecules in genetically identical cells. In these earlier studies, however, no distinction was made between transcription of the two alleles of a particular gene like mutant and wildtype as done in our study.

Yet, because of the heterozygous genotype, we can distinguish between mutant and wildtype alleles and clearly see heterogeneous expression of the two alleles among cardiomyocytes. Therefore, we set up a numerical model based on the concept of burst-like transcription, which is independent for the two alleles, mutant and wildtype. This modeling revealed a surprisingly close match with our experimental data, including cell-to-cell variation in mutant *MYH7*-mRNA and in pCa_50_, as well as the distribution of the total *MYH7*-mRNA copy number among individual cardiomyocytes (Figure 4; Figure S8). Interestingly, with stochastic, burst-like transcription of mutant and wildtype alleles, a cardiomyocyte is expected to change at the mRNA- and protein-level, once in a while, from an almost wildtype cell via a mixed cell to a cell expressing almost pure mutant protein, and vice versa (cf. Figure S8).

### How could cell-to-cell functional imbalance affect myocardial function and contribute to HCM development?

The mutations studied here, like most HCM mutations, alter force generation and Ca^++^-sensitivity of the sarcomere, consistent with the “poison peptide” mechanism (Ashrafian et al., 2011). Therefore, it seems likely that sarcomeres with high or low amounts of mutant β-MyHC generate different force levels during each twitch/heartbeat, as suggested by the heterogeneity in force generation at partial activation (Figure 1). Cardiomyocytes with low force would get distorted and stronger cardiomyocytes may over-contract. According to our model calculations, this will change with the expression of mutant β-MyHC over time (Figure S8), affecting neighboring cardiomyocytes randomly (Brenner et al., 2014). Since the myocardium is a network, where adjacent strands of serially arranged cardiomyocytes are interconnected by branched cardiomyocytes, such a functional mosaic may well contribute to the severe myocyte disarray typical for HCM (Varnava et al., 2000). Therapeutic reduction in force generation, e.g., by β-adrenergic blockers or calcium antagonists, results in a milder phenotype, as may specific small molecule inhibitors of cardiomyocyte force generation, since functional imbalance and cellular distortions will be smaller. Along these lines, diltiazem treatment of pre-clinical HCM mutation carriers suggested a delay in early left ventricular remodeling (Ho et al., 2015b).

**Figure 5:**
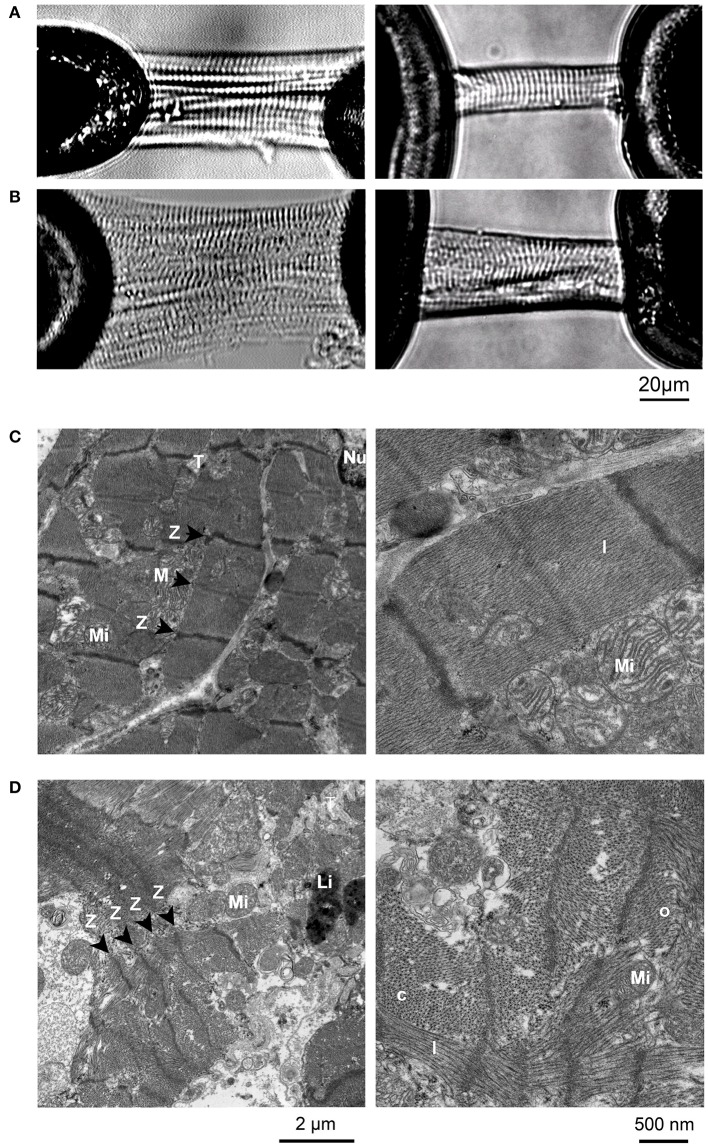
Micrographs of mounted cardiomyocytes and EM images of control and R723G samples. **(A)** Control cardiomyocytes from donor myocardium. **(B)** Cardiomyocytes with mutation R723G. Note that cardiomyocytes with mutation R723G have less well organized striation patterns than controls, consistent with myofibrillar disarray previously described for cardiomyocytes from affected HCM-patients (Kraft et al., 2013). **(C,D)** Electron micrographs of myocardial tissue from control **(C)** and patient with mutation R723G **(D)**. Different from control tissue, EM images of R723G tissue show longitudinally (l), obliquely (o), and cross sectioned (c) myofibrils right next to each other within individual cell (d, right panel) as indication of myofibrillar disarray. Z-disks (Z), M-lines (M), mitochondria (Mi), T-tubuli (T), a nucleus (Nu), and lipofuscin granula (Li) are indicated.

Even myofibrillar disarray within individual cardiomyocytes may develop since myofibrils, dependent on the time of their formation, may have quite different abundance of the mutant protein, generating subcellular functional imbalance among individual myofibrils. Electron microscopy revealed extensive disarray and loss of myofibrils in myocardium of the affected HCM patients in this (Figure 5) and previous studies (Ferrans et al., 1972; Kraft et al., 2013; Witjas-Paalberends et al., 2013). This is consistent with more disordered sarcomere structure of mutated cardiomyocytes in light micrographs (Figures 5A,B) and the observed reduced force generation per cross-sectional area of cardiomyocytes of affected patients compared to controls (Figure S5).

It should be noted that the observed heterogeneity among individual cardiomyocytes will most likely not be the only trigger of HCM-development. The globally increased or decreased myofilament calcium sensitivity caused by the mutations may well lead directly to remodeling and alterations of whole heart function, while cell-to-cell heterogeneity could exacerbate the phenotype. This is supported by findings in a severely affected homozygous HCM-patient, who developed cardiomyocyte hypertrophy and abnormal myofibrillar orientation (Nishi et al., 1994). Yet, it cannot be ruled out that an undetected additional compound heterozygous mutation may have contributed to this patient's phenotype. Overall, homozygous patients are very rare but they often show a severe and early onset phenotype that differs from that of heterozygous patients (Nishi et al., 1994; Richard et al., 2003). Similarly, homozygous mice with α-myosin- mutation Arg403Gln die within 7 days after birth, while the heterozygous animals apparently have normal life span where they develop several of the major characteristics of human HCM, including myocyte disarray (Geisterfer-Lowrance et al., 1996).

Importantly, our contractile imbalance hypothesis and the poison peptide principle do not exclude each other. Instead, the functional alterations caused by the mutated β-MyHC which is incorporated into the sarcomeres (poison peptide effect) are an essential starting point for development of the contractile imbalance. We suggest, based on our data that the fractions of functionally different mutated and wildtype β-MyHC in the sarcomeres vary from cell-to-cell due to stochastic burst-like expression of the two alleles. The resulting cell-to-cell contractile imbalance may particularly enhance progressive development of specific HCM features like structural distortions leading to cellular and myofibrillar disarray and interstitial fibrosis (Brenner et al., 2014).

## Limitations of the study

One limitation of the work presented here is that fractions of mutant vs. wildtype mRNA and contractile function cannot be studied on the same cardiomyocytes, thus precluding a direct causal relation between cell-to-cell contractile imbalance, burst-like transcription, and variation in expression of mutated β-MyHC among individual cardiomyocytes. Our functional studies require chemically permeabilized cardiomyocytes where the cytoplasm is replaced by physiological solutions with defined calcium concentrations. This precludes subsequent mRNA-isolation and analysis so that we have to rely on data from two groups of cardiomyocytes which, however, were isolated from the same pieces of cardiac tissue. In addition, the fractions of mutant vs. wildtype myosin protein cannot be studied at the single cell level since this requires a quantitative mass spectrometry approach with single cell sensitivity which is not available. Instead we suggest that the large functional variability (e.g., of pCa curves) of the cardiomyocytes is an indirect measure for cell-to-cell variability of mutated protein in the sarcomeres.

We also cannot exclude that post-translational modifications of contractile proteins which may even vary from cell-to-cell contribute to the observed decrease in Ca^++^-sensitivity and the functional heterogeneity among individual mutated cardiomyocytes. However, the increased heterogeneity in HCM-patients as compared to donors was detected even though at least PKA-dependent phosphorylation levels were adjusted for all cardiomyocytes by treatment with PKA.

The data presented here from three HCM-patients provide experimental evidence for highly variable expression of β-MyHC-mutations in two male patients at end stage of the disease (R723G) and in a female patient (A200V) undergoing myectomy at the age of 19 years (see Supplementary Material). Despite the limitation to three patients the data indicate, however, that the variable allelic expression from cell-to-cell is not associated with the end stage situation of the HCM-heart. Yet, whether the proposed concept of independent, stochastic, burst-like transcription of the two alleles as trigger for HCM does also apply to *MYH7*-mutations that cause an increase in calcium-sensitivity and to HCM-mutations in other sarcomeric proteins remains to be elucidated in further studies.

## Conclusion

Our data show a significant cell-to-cell variation in mutated vs. wildtype *MYH7*-mRNA-expression and contractile properties among individual cardiomyocytes isolated from the same tissue samples of HCM patients with two different β-MyHC-mutations. This large variability at the mRNA-level is most likely due to stochastic, burst-like *MYH7*-transcription, which is independent for the mutant and the wildtype allele, leading to a similarly large cell-to-cell variability in mutated vs. wildtype protein. The resulting large variation in contractile properties among individual cardiomyocytes may contribute to cellular and myofibrillar disarray and, by distortion of non-cardiomyocytes, trigger stretch sensitive signaling paths that lead to development of interstitial fibrosis. We hypothesize that such stochastic, burst-like transcription of the related alleles may also induce similar changes for other heterozygous HCM mutations.

## Author contributions

BB, TK, JM, and KK designed experiments. TK, JM, KK, MM, PE, JB, EB, ST, BK, and CM performed experiments. AR and BB set up and performed model simulations. KW, APi, JvdV, CdR, APe, AF and FN-L provided experimental setups or acquired tissue samples. BB, TK, JM, and AR wrote the manuscript with contributions from all authors.

### Conflict of interest statement

The authors declare that the research was conducted in the absence of any commercial or financial relationships that could be construed as a potential conflict of interest.
