# Supplementary material for: Burst-Like Transcription of Mutant and Wildtype MYH7-Alleles as Possible Origin of Cell-to-Cell Contractile Imbalance in Hypertrophic Cardiomyopathy
Source: Front Physiol. 2018 Apr 9;9:359. doi: 10.3389/fphys.2018.00359 (PMC5900384; doi:10.3389/fphys.2018.00359)
Supplement: Supplementary file 1 [file Presentation1.pdf]

Supplementary Material

Burst-like transcription of mutant and wildtype *MYH7*-alleles as possible origin of cell-to-cell contractile imbalance in hypertrophic cardiomyopathy

*Judith Montag, Kathrin Kowalski, Mirza Makul, Pia Ernstberger, Ante Radocaj, Julia Beck, Edgar Becker, Snigdha Tripathi, Britta Keyser, Christian Mühlfeld, Kirsten Wissel, Andreas Pich, Jolanda van der Velden, Cristobal G. dos Remedios, Andreas Perrot, Antonio Francino, Francesco Navarro-López, Bernhard Brenner, Theresia Kraft**

** Correspondence: Dr. Theresia Kraft:* Kraft.Theresia@mh-hannover.de

1 Supplemental Materials and Methods

Patients and controls

The study on anonymized human tissue was approved by the Ethics Committee of Hannover Medical School (No. 2276-2014). Informed consent was obtained according to approved Ethics Committee protocols of the institutions involved. The investigations conformed to the principles of the Declaration of Helsinki (WMA, 1997).

Patient with β-MyHC-mutation p.A200V

This female patient was severely affected by HCM and showed an early disease onset. She was diagnosed with hypertrophic obstructive cardiomyopathy (HOCM) and a myectomy was performed at the age of 19 when the septum thickness had gained 33 mm (posterior wall thickness 16 mm) with a left ventricular outflow tract gradient of 100 mmHg, fractional shortening was 50%, and she was NYHA class II. The septum tissue sample was shock frozen in liquid nitrogen immediately after the myectomy and stored in liquid nitrogen until further use. Genotyping of 24 HCM associated genes revealed the point mutation c.599C>T in β-MyHC, resulting in an exchange of alanine vs. valine at position 200 (p.A200V).

Patients with β-MyHC-mutation p.R723G

Samples of ventricular myocardium of two male HCM patients became available when they received a heart transplant at the age of 55 and 38, respectively. The patients are members of two families from Barcelona, their clinical characteristics were published previously ((Enjuto et al., 2000; Kraft et al., 2013); patient II-5 of family 26, and patient III-1 of family 157). Both had developed the clinical phenotype of HCM with hypertrophy of the interventricular septum and the left ventricular wall. They showed ECG- irregularities including ST-T wave abnormalities; patient II-5 received an implantable cardioverter-defibrillator several years

before heart transplantation. The analysis of 24 HCM-associated genes revealed the previously described mutation c.2167C>G in the ventricular β-myosin heavy chain (β-MyHC), resulting in an exchange of arginine vs. glycine at position 723 (p.R723G). Tissue samples of interventricular septum, lateral as well as inferior posterior ventricular wall of the explanted heart were shock frozen in liquid nitrogen immediately after dissection. mRNA and functional data obtained with cardiomyocytes from the different cardiac regions of the R723G patient were very similar and therefore the data were combined for further analysis.

Controls

Flash-frozen control heart tissue of the left-ventricular free wall and the interventricular septum was from non-transplanted donor hearts for which no suitable recipient had been found (n=5, 23–52 years of age, mean age 41±11 years; 1 female, 4 males). The donor heart tissue samples were obtained from the Sydney Heart Bank, University of Sydney, Australia. Previously it was shown for control cardiomyocytes that functional parameters of sarcomere contraction as they are measured here are not age-dependent (Hamdani et al., 2010).

Frozen tissue samples

Cardiac tissue samples frozen in liquid nitrogen were split into smaller pieces by gently crushing the tissue with a cooled pestle in a liquid nitrogen-cooled mortar. These frozen pieces were then used for mRNA quantification and to isolate cardiomyocytes for functional studies.

Force measurements of single cardiomyocytes

Cardiomyocytes were mechanically isolated and chemically permeabilized as previously described (Kraft et al., 2013). From the cardiomyocyte suspension, a single, cylindrically shaped cardiomyocyte showing striations was selected and attached to a motor (High Speed Length Controller, Aurora Scientific, Canada) and force transducer with silicone adhesive. The selection of cardiomyocytes suitable for mechanical measurements followed the same criteria (cell length, cell width, striation pattern) for the donors and the HCM-patients' cardiomyocytes. Sarcomere length of the mounted cardiomyocytes was adjusted to 2.2 µm. Cardiomyocytes with mutation R723G were from inferior/posterior left ventricular wall (n=10; open red symbols in Figure 1) and from interventricular septum (n=12; filled red symbols in Figure 1), the respective donor cardiomyocytes were from the left ventricular wall of four different donors. Due to the small inter-sample variability of the pCa-curves of the donor cardiomyocytes the data were pooled. Cardiomyocytes with mutation A200V and respective donor cardiomyocytes were from the interventricular septum.

Force generation was measured in activating solutions of different Ca^{++}-concentrations which are shown as pCa-values ($-_{10}\log[Ca^{++}]$), as described previously (Kraft et al., 2013). Values ranging from relaxing conditions (pCa 9.0) to saturating Ca^{++}-concentration (pCa 4.63) were chosen. Upon transfer of the mounted cardiomyocyte from a trough with relaxing solution to a trough with an activating solution force started to develop. For cardiomyocytes with mutation A200V, a protocol as shown in Figure S1 was applied, with several short releases of

the cardiomyocyte during the initial phase of Ca^{++} activation. This allowed stabilization of the striation pattern while Ca^{++} could equilibrate (Brenner, 1983). For cardiomyocytes with mutation R723G, the experimental protocol was as shown previously (Kraft et al., 2013). In all cases, once force had reached a steady state level, a short length-release was applied to slacken the cardiomyocyte within 1ms by 40% of its initial length. This allowed free shortening of the cardiomyocyte under zero load and recording of the zero force level. Total force (F_{tot}) was determined as the difference between steady state force and zero force level. After several ms of free shortening the cardiomyocyte was set back to its original isometric length by the motor, and force could redevelop back to the original isometric steady state level (force redevelopment). Then the cardiomyocyte was rapidly moved back to relaxing solution. After the cardiomyocyte had fully relaxed, the cardiomyocyte was slackened for 10 sec by a length release (30% of cell length) to determine passive force (F_{pass}). Active force was calculated as $F_{act}=F_{tot}-F_{pass}$. To record a force-pCa-relation, this procedure was repeated with activating solutions of different concentrations of free Ca^{++}-ions. The sequence of Ca^{++}-concentrations was randomized. The time for force redevelopment was adjusted so that for all activation levels steady state force was reached. At the end of each pCa-series another maximum activation was carried out to determine the force run down of the respective cardiomyocyte by comparison this value to the initial maximum force value. For donors and HCM-patients cardiomyocytes with more than 20% force rundown were excluded from the analysis.

Solutions for R723G-cardiomyocytes were as described previously (Kraft et al., 2013). For A200V-cardiomyocytes the relaxing solution contained (in mM): 10 imidazole, 2 $MgCl_2$, 3 EGTA, 10 CrP, 10 caffeine, 2 MgATP, 110 K-propionate; pCa about 9; the activating solution contained (in mM): 10 imidazole, 2 $MgCl_2$, 3 CaEGTA, 10 CrP, 10 caffeine, 2 MgATP, 110 K-propionate; pCa 4.5. The pH of both solutions was adjusted to 7.1 at 15°C by adding the appropriate amount of KOH. Chemicals were from Sigma-Aldrich (Steinheim, Germany). Relaxing and activating solutions were mixed in different proportions to obtain activating solutions of different pCa values. The proportions required for different pCa-values were calculated using the program "calcium" (Fohr et al., 1993). To minimize variability arising from day to day solution mixing, activating solutions with different pCa values were pre-mixed in larger quantities, divided in small aliquots and kept frozen at -20°C until use.
Since previous work showed that PKA-dependent phosphorylation in donor and patient's myocardium can be quite different (van der Velden et al., 2003; Kraft et al., 2013), we aimed to exclude effects of different PKA-dependent phosphorylation on our mechanical measurements. Therefore, force measurements were performed after a 1hr incubation of the mounted cardiomyocyte with Protein Kinase A (PKA, Sigma-Aldrich, Steinheim, Germany) at 20°C to maximize phosphorylation of PKA-dependent sites, particularly of cardiac troponin I (cTnI). Based on gel electrophoretic analysis at the tissue level such treatment yields comparable PKA-dependent phosphorylation of donor cardiomyocytes and cardiomyocytes of the HCM-patients (Kraft et al., 2013).
To compare absolute forces generated by the cardiomyocytes, the observed F_{act} at saturating Ca^{++}-concentration was related to the cross sectional area of the cardiomyocyte. To determine the cross-sectional area, the width of the mounted cardiomyocyte was measured with the light

microscope in the setup (Axiovert 100, Zeiss, Oberkochen, Germany) with a 32x objective lens. The height was measured by imaging the cardiomyocytes through a small prism placed parallel to the axis of the mounted cardiomyocyte (20x objective lens) (Belin et al., 2006). The cross-sectional area was calculated assuming an elliptically shaped cross-section.

Note that the protocol for force measurements and the solution composition for R723G and A200V experiments were slightly different, while conditions otherwise (e.g., experimental temperature, PKA-incubation) were identical. However, the differences of solutions and protocol had no substantial impact on the force-pCa-curves. This can be seen e.g., from the highly similar pCa_{50} values of donor cardiomyocytes from the two sets of experiments in Figure 1c and 1d.

Quantification of mutant vs. wildtype *MYH7*-mRNA in individual cardiomyocytes

Isolation of individual cardiomyocytes by laser microdissection

Flash frozen cardiac tissue samples were mounted on the sample plate of a cryomicrotome (Microm HM 560; Thermo Fisher Scientific, Schwerte, Germany) with OCT and sections of 5 µm thickness were cut from the non-embedded side. The sections were mounted on slides coated with a PEN-membrane (Zeiss, Oberkochen, Germany) and fluorescently stained for cadherin. Before staining the sections were fixed in ice-cold 75% ethanol and washed for 10 s in ice cold PBS. For the staining of intercalated discs, the tissue sections were incubated for 3 min at room temperature with 15 µl of the primary anti-cadherin antibody (Abcam,) diluted 1:10 in PBS with 2mg/ml BSA. The slides were washed twice for 10 s in ice cold PBS. This was followed by incubation for another 3 min at room temperature with 15 µl of the secondary anti-rabbit-TRITC antibody (Sigma-Aldrich, Steinheim, Germany), also diluted 1:10 in PBS with 2 mg/ml BSA. This was followed by three washes, 10 s each, with ice cold PBS. Tissue sections were then dehydrated in ice cold ethanol (75%, 95%, 100%, for 10 sec, 30 sec, and 1 min, respectively).

Single cardiomyocytes were isolated by laser microdissection (LMD) with a Zeiss PALM setup. Cardiomyocytes were identified by fluorescently labeled cadherin in the desmosomes and the striation pattern in bright field illumination (Figure S2). Individual cardiomyocytes were marked, laser dissected, and catapulted into 27 µl nuclease free water in the lid of a PCR-tube. Cardiomyocytes were lysed by one freeze-thaw cycle (freezing in liquid nitrogen and thawing at room temperature) and subsequent incubation at 75°C for 10 min.

Quantitative single cell RT-PCR

The total volume of 27 µl of each lysate from single cardiomyocytes was mixed with 13 µl reverse transcription reaction mix (1x reaction buffer, 0.125 mM dNTPs each, 0.4 µM *MYH7* specific primers (Table S1), 1 U/µl RNase inhibitor (RiboSafe, Bioline, Luckenwalde, Germany), and 5 U/µl reverse transcriptase (Tetro RT, Bioline)). The sample was mixed using a micromixer for 10 min at room temperature (Boon et al., 2011). For subsequent cDNA-synthesis samples were split into two aliquots of 20 µl each. Both aliquots were incubated on the micromixer for 1 h at 42°C.

For relative quantification of the *MYH7* alleles in the single cardiomyocytes, a nested PCR was applied. For the first PCR, the complete cDNA was amplified in PCR reaction mix that

contained 1x reaction buffer, 0.5 mM $MgCl_2$, 0.2 mM of each dNTP, 0.2 µM of both forward and reverse nested primers 1 (Table S1), and 0.04 U/µl HotStarTaq (Qiagen, Hilden, Germany), final volume 100 µl. Initial activation was performed for 15 min at 95°C. Subsequently, 45 cycles were applied with 95°C for 30 sec, 67°C (R723G) or 60°C (A200V) for 30 sec, and 72°C for 30 sec. The final elongation was performed at 72°C for 2 min.

For the second, nested PCR, 1 µl of the PCR-product was transferred to the PCR reaction mix that contained 1x reaction buffer, 0.5 mM $MgCl_2$, 0.2 mM of each dNTP, 0.2 µM of both forward and reverse nested primers 2 (Table S1), and 0.04 U/µl HotStarTaq (Qiagen, Hilden, Germany) in a final volume of 25 µl. Following an initial activation for 15 min at 95°C, 45 cycles were applied with 95°C for 30 sec, 67°C (R723G) or 62°C (A200V) for 30 sec, and 72°C for 30 sec. The final elongation was performed at 72°C for 2 min. To ensure that during first and second PCR both, wildtype and mutant mRNA were amplified, as control a defined 50/50 mixture of standard plasmids that encoded for the wildtype and mutated R723G sequence of the respective PCR amplicons were run in parallel through the same protocol.

To account for possible heteroduplex-formation, a reconditioning PCR was performed (Thompson et al., 2002). For this 2.5 µl of the second nested PCR were transferred to a final volume of 25 µl PCR reaction mix identical to the second nested PCR, and the respective PCR protocol was run for three successive cycles.

Allele specific restriction digest

12.5 µl of the reconditioned PCR products were treated with *Mbo*I or 5 U *Hpy*4CHI (both New England BioLabs, Ipswich, MA, USA) for the mutation R723G or A200V, respectively, in a final volume of 15 µl in the respective restriction buffer for at least 3 h at 37°C. For mutation R723G, the digest resulted in a 145 bp band for both alleles, a 125 bp and a 90 bp fragment for the mutant and the wildtype allele, respectively (Kraft et al., 2016). For mutation A200V, the digest yielded a 85 bp fragment for both alleles, a 108 bp and 138 bp fragment specific for the mutant and wildtype mRNA, respectively (Figs. 2A, 2B and S3A).

Quantification of allelic expression

*Mbo*I or *Hpy*4CHI-treated PCR products were separated on 3.5% sieving agarose gels stained with ethidiumbromide (Figure 2A, Figure S3A; for R723G see (Kraft et al., 2016). Quantification of mutant vs. wildtype *MYH7*-mRNA was performed as described previously in detail (Tripathi et al., 2011). In brief, the restriction fragments were analysed densitometrically using the TotalLab (Newcastle upon Tyne, Great Britain) and Origin (OriginLab, Northampton, MA, USA) software, yielding the integrated optical density (IOD) of each band. The IOD was normalized against the number of base pairs to correct for the concentration of intercalated stain. The fraction of mutant *MYH7*-mRNA was calculated as the ratio of IOD/bp of the mutation-specific fragment over IOD/bp of the fragment generated by both mutant and wildtype *MYH7*-mRNA.

Control of linearity

For both mutations, we tested the linearity of our quantification procedure as previously described (Tripathi et al., 2011) using standard plasmids that encoded for the wildtype, the R723G, or A200V sequence of the respective PCR amplicons. These were mixed in defined

ratios (20/80; 40/60; 50/50; 70/30; 90/10) and used as templates for qPCR, reconditioning PCR and the subsequent allele specific restriction digest. Quantification of the restriction digest of the standard mixtures revealed that the mixed allelic templates were amplified and detected in direct proportion to the stoichiometric fraction of each template (Figure S3B,C for mutation A200V; for mutation R723G cf. Tripathi et al. (Tripathi et al., 2011)). Thus, within the range of our tested mixtures of standard plasmids we can retrieve the fraction of mutant or wildtype *MYH7*-mRNA with high accuracy (error <4.4%).

Quantification of experimental scatter

To determine the experimental scatter of our single-cell quantification method, we performed parallel quantification of mutant vs. wildtype mRNA from a diluted larger mRNA sample. A section of R723G septum tissue was lysed and diluted serially and subjected to quantitative single cell RT-PCR. The integrated optical density of PCR products of these dilutions and of single cardiomyocytes were determined and normalized to the 300 bp band of the equimolar standard ladder on each gel (4 µl/lane; Carl Roth, Karlsruhe, Germany). The diluted lysate with a normalized IOD that was comparable to that of single cardiomyocytes was then divided into 13 aliquots. These aliquots were subjected to parallel, quantitative single cell RT-PCR as described for single-cell quantification of mutated and wildtype mRNA, with subsequent allele specific restriction digest and densitometric quantification (Figure 2D).

Absolute quantification of the *MYH7*-mRNA copy number in individual cardiomyocytes

Standard-RNA was generated by in vitro transcription using *MYH7*-cDNA (clone IRCMp5012C0225D, Source BioScience, Berlin, Germany) as a template for the T7-MegaScript Kit (Thermo Fisher Scientific, Schwerte, Germany) according to the suppliers' instructions. Standard-RNA was isolated as described above and the concentration was determined using a NanoDrop device (PeqLab, Erlangen, Germany).

For the absolute quantification of *MYH7*-mRNA in individual cardiomyocytes, cells were microdissected from R723G cardiac tissue slices (5µm thickness) as described above and lysed. The single-cell lysate and serial dilutions of the standard-RNA of $1x10^6$, $1x10^5$, $1x10^4$, $1x10^3$, and $1x10^2$ copies were reverse transcribed in parallel in a final volume of 40 µl reverse transcription reaction mix (1x reaction buffer, 0.125 mM dNTPs each, 0.4 µM primer Exon16_rev (Table 1)), 1 U/µl RNase inhibitor (RiboSafe, Bioline, Luckenwalde, Germany), and 5 U/µl reverse transcriptase (Tetro RT, Bioline). The cDNA was then subjected to a pre-amplification PCR using 1x TaqMan Master Mix (Applied Biosystems, Foster City, CA, USA), 0.4 µM of each forward and reverse real-time PCR primer (Table S1) in a final volume of 100 µl. PCR was performed after initial denaturation at 95°C for 10 min, followed by 40 cycles of 95°C for 45 sec and 60°C for 45 sec. Real-time PCR was performed in a final volume of 20 µl. 1 µl of pre-amplified PCR product was transferred to 1x TaqMan Master Mix, 0.4 µM of each, real-time PCR probe and forward and reverse real-time PCR primer, respectively (Table S1). After initial denaturation at 95°C for 10 min, 40 cycles of 95°C for 45 sec and 60°C for 45 sec were applied using an ABI 7500 Fast Real-time PCR setup (Applied Biosystems, Foster City, CA, USA). The copy number per cardiomyocyte was calculated from linear regression analysis of the standard RNA.

Quantification of the fraction of mutated protein in tissue samples with β-MyHC-mutation A200V

Quantification of mutated and wildtype protein in myocardial tissue with mutation A200V was performed according to the same mass-spectrometry approach as previously published for other β-MyHC-mutations (Becker et al., 2007). Briefly, sarcomere-bound myosin was extracted from fragments of a myectomy sample with mutation A200V. The myosin was subjected to a trypsin digest with an enzyme to myosin ratio of 1:27. Myosin was digested for 2x24 hrs at 37°C on a shaker, with adding the same amount of trypsin after the first 24 hrs. The buffer for digestion was 40mM ammonium bicarbonate (Sigma Aldrich, Steinheim, Germany), pH 8.0 in 10% acetonitrile (ACN; LiChrosolv/Merck, Darmstadt, Germany) in 90% H_2O (Milli-Q Synthesis A10, Merck, Darmstadt, Germany). Trypsin was from Promega Corporation, Madison, USA (Mass Spectrometry Grade). After digestion, the myosin peptide mix was lyophilized and redissolved in 20% ACN, 0.1% TFA (trifluoroacetic acid; J.T. Baker, Griesheim, Germany), and 1% DMSO (Sigma-Aldrich, Steinheim, Germany) and stored at minus 20°C until analysis.

For quantification of mutated and wildtype β-MyHC, the 14 amino acid peptide generated by the trypsin digest was used that contained the mutation site (at position 10) with an alanine or valine in the wildtype and mutant peptide, respectively (Table S2). As internal quantification standards equal quantities of synthetic stable-isotope-labeled forms of the mutant and wildtype 14 amino acid peptide (Table S2), respectively, were added before myosin was digested. These standard peptides were synthesized with a purity of more than 98% and a peptide content of 89% (verified by quantitative amino acid analysis; Coring System Diagnostix GmbH, Gernsheim, Germany). The peptides were dissolved in H_2O (Milli-Q Synthesis A10, Merck, Darmstadt, Germany) with (v/v) 35% ACN, 0.1% TFA and 10% DMSO. DMSO was added to ensure a complete dissolution of the lyophilized isotope-labeled peptides. In further dilution steps DMSO was reduced to a concentration of less than 5%.

The peptide mix of the myosin digest was directly applied to a HPLC-system (Agilent 1100 Capillary Pump System; Agilent Technologies, Waldbronn, Germany) connected to an electrospray mass spectrometer (Bruker Esquire3000 plus; Leipzig, Germany); i.e., different from our previously published method (Becker et al., 2007) the peptide mix was not pre-fractionated. Injection volume of the peptide mix including the internal standard peptides was 1µl. The separation column (Agilent Zorbax 300 SB-C18, 5 µm, 150 x 0.5 mm) was run with a flow rate of 5 µL/min. Peptides were separated with an increasing ACN-gradient starting at 5% ACN with 0.1% TFA in H_2O and ending at 80% ACN with 0.1% TFA. Quantification of the target peptide ions was based on extracted ion chromatograms (Figure S4) with a width of m/z±0.5 using the software Bruker Daltronics QuantAnalysis 1.6.

Fluorescence in situ hybridization (FISH) for visualization of active transcription sites (*MYH7*-pre-mRNA) and cytoplasmic *MYH7*-mRNA

To visualize active transcription sites by fluorescence in situ hybridization (FISH) we labelled pre-mRNA by two Stellaris® probe sets (BioSearch Technologies, Petaluma, CA, USA). One set contained 48 of 20-mer oligonucleotides designed to label intronic sequences of *MYH7*, the other contained 48 of 20-mer oligonucleotides to label exonic sequences. Of the first set

each oligonucleotide was labelled with one Quasar 670 fluorophore, of the second set each oligonucleotide was labelled with one Quasar 570 fluorophore.

Our hybridization procedure followed the protocols of BioSearch Technologies (Petaluma, CA) and of (Lyubimova et al., 2013) with some modifications. First, frozen tissue samples were pre-fixed in 4% paraformaldehyde (PFA) at 4°C for 5-6h. Next, the samples were completely embedded in Tissue-Tek O.C.T. (Sakura Finetek Europe, Leiden, NL) on dry ice and stored in liquid nitrogen until cryo-sectioning. The cryo-sections (5µm or 16µm thick) were attached to HistoBond® adhesive mircoscope slides (Marienfeld GmbH & Co.KG, Lauda-Königshofen, Germany) and fixed for 20 min with 4% PFA (in PBS) at room temperature. Subsequently the sections were washed three times with 1x PBS (w/o Mg^{2+}, Ca^{2+}). Then the tissue sections were permeabilized for 1.5h in 70% EtOH at 4°C and incubated in wash buffer (10% formamide, 2x saline-sodium citrate (SSC) in nuclease-free water) for 2-5min. This was followed by the over-night hybridization. For hybridization, tissue sections were surrounded by a Teflon ring attached to the cover slips by grease to minimize the necessary volume of hybridization buffer. The hybridization buffer contained 125nM of each probe set (Lyubimova et al., 2013). The hybridization was done in a sealed humidified chamber at 37°C with a 13mm cover slip on top of the buffer-filled Teflon ring to prevent evaporation. After hybridization, samples were washed twice for 30min with wash buffer (see above) at 37°C. In the second wash DAPI was added at a concentration of 40ng/mL. Next, the samples were incubated in 2xSSC for 2-5min at RT. After removing teflon ring and vacuum grease, 13µl GLOX anti-fade buffer was added before imaging (Lyubimova et al., 2013).

Samples were imaged with an Olympus IX83 fluorescence microscope with a 60x oil objective (ApoN TIRFMN.A. 1.49, Olympus, Tokyo, Japan) and a metal halide light source. Images were recorded with a cooled CCD camera (Orca-R^2, Hamamatsu, Photonics, Japan). Three-dimensional z-stacks were recorded with motorized shutter and z-stage using filter sets for DAPI (Chroma U-F4900, Chroma Technology Corp, Bellows Falls VT, USA), GFP (Chroma U-F49002), Cy3 (Chroma U-F49004) and Cy5 (Chroma U-F49006). Exposure times for thick sections (16µm) were 40ms for DAPI, 350ms for GFP and Quasar 570, and 1s for Quasar 670. For thin sections (5µm) we used 40ms for DAPI, 400ms for GFP and Quasar 570, and 1s for Quasar 670. Adjacent images in z-stacks were separated by 0.3µm.

The number of active transcription sites, i.e., spots with double fluorescence of both probe sets inside nuclei, were counted manually using cellSens Dimension (version 1.13, Olympus, Tokyo, Japan).

Estimate of ploidy of nuclei of cardiomyocytes

Cardiomyocytes can be polyploid, in normal and in hypertrophied myocardium (Brodsky et al., 1994; Herget et al., 1997). It was shown that the volume of DAPI-stained nuclei is proportional to their ploidy (Bergmann et al., 2011; Bahar Halpern et al., 2015). To determine the ploidy of nuclei in the R723G-sample we determined the largest cross-sectional area of DAPI-stained nuclei in z-stacks recorded from frozen tissue sections of 16µm thickness. The largest cross sections of 638 nuclei located inside cardiomyocytes and of 774 nuclei located outside cardiomyocytes were determined using Olympus imaging software (cellSens Dimension version 1.13, Olympus, Tokyo, Japan). The nuclei outside cardiomyocytes, e.g., of

cells in connective tissue, were taken as the reference for diploid (2n) nuclei. An estimate of the volume of the nuclei was obtained by calculating the 3/2 power of the largest cross-sectional area. This way of volume estimate was performed since not all nuclei were fully enclosed even in 16µm sections. From the volume distribution of the 774 nuclei outside cardiomyocytes we estimated the mean volume of 2n nuclei by maximum likelihood fitting of their volume distribution with a Gaussian function. The thus obtained mean volume of 150 μm^3 was taken as the initial estimate for maximum likelihood fitting of the volume distribution of the 638 nuclei inside cardiomyocytes. Gaussian functions with means that were multiples of the 2n volume were used, corresponding to different ploidy levels (2n, 4n, 8n, 16n, and 32n). The SD of the Gaussians was set proportional to the mean volume, i.e., proportional to ploidy, using the SD obtained from the fitting of the 774 nuclei outside cardiomyocytes (2n). As a result we found for the R723G cardiac tissue 50.4% diploid, 17.6% tetraploid, 7.1% octoploid, and 11.3% 16-ploid and 13.5% 32-ploid cells. These values were used for the model calculations described below. For comparison, in donor myocardial tissue 19.3% of the cardiomyocytes were diploid, 45.4% tetraploid, 23.1% octoploid, 11.3% 16-ploid and 0.9% 32-ploid, which corresponds to similar values for normal heart tissue in the literature (Herget et al., 1997).

Modelling of cell-to-cell variation in mutant mRNA and mutant protein by burst-like, independent, stochastic transcription of the mutant and wildtype allele

The model calculations were based on stochastic, burst-like transcription (Raj et al., 2006). Previously, modelling of burst-like transcription was done to account for large variation in mRNA or protein expression in individual cells of a genetically homogenous cell population (Raj et al., 2006). Different from this previous modelling, here we distinguish transcription and translation of the two alleles, mutant and wildtype, in terms of mutant vs. wildtype *MYH7*-mRNA as well as mutant vs. wildtype β-MyHC-protein in individual cardiomyocytes. We can compare the model predictions with the *in vivo* situation since we have experimental information about mutant vs. wildtype transcript levels and (indirectly via function) about mutant vs. wildtype protein of the two β-MyHC (*MYH7*) alleles from our study on heterozygous point mutations in the *MYH7* gene, e.g., mutation R723G.

We assume stochastic, independent, burst-like transcription of the two alleles of the *MYH7* gene. To model independent, burst-like transcription and translation of the two alleles, we set up the differential equations for both alleles with identical kinetic parameters (Table S3). Only the degradation rate of mutant mRNA with the R723G mutation was set to half of that of wildtype *MYH7*-mRNA to account for the approximately 2:1 average fraction of mutant:wildtype mRNA and protein in tissue samples of R723G patients (Figure S8a (Tripathi et al., 2011)). This is based on preliminary evidence that mutation R723G affects the mRNA-stability (own, unpublished observation).

The relevant reactions were (i) the stochastic opening and closing of the transcription sites described by the rate constants k_act and k_inact, (ii) the transcription process to pre-mRNA with the rate constant k_plus_pre-mRNA, (iii) generation of mRNA by splicing (rate constant k_plus_mRNA) and mRNA degradation with the rate constant (k_minus_mRNA), and (iv) the translation into protein and protein degradation with the rate constants for protein synthesis per mRNA molecule and protein degradation (k_plus_Protein, k_minus_Protein,

respectively). Thus, each iteration run consisted of solving the following four equations sequentially and for each of the two alleles separately:

```
DNA[i] <- (1-DNA[i-1])*Rbin_akt[i-1]+DNA[i-1]*(1-Rbin_inakt[i-1])
premRNA[i] <- premRNA[i-1] + DNA[i]*k_plus_premRNA - premRNA[i-1]*k_plus_mRNA
mRNA[i] <- mRNA[i-1] + premRNA[i]*k_plus_mRNA - mRNA[i-1]*k_minus_mRNA
Protein[i] <- Protein[i-1] + mRNA[i]*k_plus_Protein - Protein[i-1]*k_minus_Protein
```

Thereby, Rbin_akt and Rbin_inakt are vectors consisting of zeroes and ones with the probability for ones being k_act and k_inact, respectively. DNA is a vector representing the open (1) or closed (0) state of the transcription site at each iteration point [i].

DNA produces pre-mRNA with a specified synthesis rate (k_plus_pre-mRNA) and pre-mRNA is directly transformed into mRNA by splicing. Accordingly there is no degradation of pre-mRNA, but mRNA is produced with the same rate as pre-mRNA is diminished (premRNA[i] *k_plus_mRNA).
The model was set up to accommodate different proportions of di-, tetra-, octo- and higher-ploid nuclei in the following way: The user defined number of iterations was split in five parts according to the proportion of di-, tetra-, octo-, 16- and 32-ploid nuclei. For the first of the five parts the simulation was done with a diploid model, for the second part with a tetraploid model, for the third part with an octoploid model, and so on. Thereby, for the diploid model only one simulation run was performed each for the mutant and the wildtype allele. For the tetraploid model two simulation runs were performed simultaneously each for the mutant and wildtype allele. For the octoploid model four simulation runs were performed simultaneously each for the mutant and wildtype allele, and so on. Time courses of on/off switching of transcription sites, mutant and wildtype pre-mRNA, mutant and wildtype mRNA, fraction of mutant mRNA, and of mutant protein were plotted individually for di-, tetra-, octo-, 16- and 32-ploid cells (cf. Figure S8 showing time courses for di- and tetraploid cell). Thus, frequency distributions of the simulated variables (mutant and wildtype pre-mRNA counts, mutant and wildtype mRNA counts, fraction of mutant mRNA and fraction of mutant protein) for all five parts put together are representative for the properties of a multitude of cells.

To be able to compare the model outcome to experimentally observed active transcription sites in nuclei (by spots with fluorescently labelled pre-mRNA), for every iteration run the existence of one or more pre-mRNA molecules was determined and counted. This was done for both alleles in the diploid proportion, four alleles in the tetraploid proportion, eight alleles in the octoploid proportion, etc. The resulting distribution of the pre-mRNA count (0, 1, 2, 3, etc.) was compared to the measured distribution of the active transcription site count.

Constraints for the model

(i) The first constraint was the experimentally observed cell-to-cell variation in mutant mRNA for R723G (mean and width of distribution, Figure 2), (ii) the second constraint was cell-to-cell functional variation, e.g., of the pCa_{50} value for R723G (mean and width of distribution, Figure 1), as indirect evidence for cell-to-cell variation of mutant protein. (iii) The modelling

had to account for the median and distribution of the *MYH7*-mRNA copy number per cardiomyocyte (mutant plus wildtype) seen with absolute quantification (Figure 3). (iv) The modelling had to account for the observed fraction of nuclei without active transcription sites (27%) (Figure 3A). (v) The modelling was further constrained by the total number of β-MyHC-protein molecules expected for cardiomyocytes. This was estimated for a 5x10x50 μm^3 cardiomyocyte from the known number of myosin molecules per myosin filament, the myosin filament packing revealed by X-ray diffraction, a sarcomere length of 2.2µm, and considering that half of the cell volume is occupied by myofibrils in tissue samples of HCM-patients (Kraft et al., 2013). This estimate yielded a total number of about $2x10^8$ myosin molecules per cardiomyocyte. (vi) A further constraint was the estimated mRNA degradation rate (k_minus_mRNA) for the *MYH7*-mRNA in differentiating mouse embryonic stem cells (Sharova et al., 2009) and the degradation rate of mRNA of various myosins in mouse fibroblasts (Schwanhausser et al., 2011) in the order of 0.06-0.09/h. (vii) The transcription rate constant (k_plus-pre-mRNA) was constrained by the observed total number of mRNA copies per cardiomyocyte (median = 589; Figure 3). (viii) The rate constant for protein synthesis per *MYH7*-mRNA molecule could be estimated from the fractional synthesis rate of β-MyHC in rabbits (Everett et al., 1983) and the median *MYH7*-mRNA copy number per cardiomyocyte (Figure 3), yielding a rate constant for translation around 2400 protein molecules per mRNA molecule per hour. (ix) The order of magnitude for the degradation rate for β-MyHC-protein was derived from the half-life measured for myosins in mouse fibroblasts, about 0.012/h (Schwanhausser et al., 2011). The only adjustable parameters in our modelling were the activation/inactivation of transcription of the two alleles (k_act, k_inact) and the splicing rate constant from pre-mRNA to mRNA (k_plus_mRNA).

Results from modelling

The rate constants are shown in Table S3. Figure S8 shows a time-period of 80 days of a predicted time course of different model output variables for an individual diploid and individual tetraploid cardiomyocyte, each heterozygous for β-MyHC-mutation R723G. The on/off-state of transcription of the two alleles, the copy numbers of mutant and wildtype *MYH7*- pre-mRNA and -mRNA molecules, the fraction of mutant *MYH7*-mRNA, as well as the fraction of mutant β-MyHC are illustrated.

To account for our observed cell-to-cell variation in mutant *MYH7*-mRNA, including cardiomyocytes with essentially pure mutant or pure wildtype *MYH7*-mRNA (cf. Figure 2c), qualitatively the pauses between transcription bursts, at least once in a while, have to be long enough for substantial decay of mRNA before the next burst (Figure S8). As a consequence, at the moment of freezing the tissue sample, in some of the cardiomyocytes mRNA is present only from one allele, the wildtype or the mutant. Yet, because of the longer life time of the protein compared to the mRNA (Table S3), the resulting bursts of mRNA are smoothed out at the protein level to a certain extent (Figure S8), depending on the lifetime of the related protein relative to the lifetime of mRNA (see also Raj et al., 2006).

To be able to directly compare the results of 35 individual cardiomyocytes flash frozen and microdissected from myocardial tissue to the outcome of the simulation (fractions of mutant *MYH7*-mRNA and mutant β-MyHC protein), we randomly picked 35 points of a very long (10,000,000 time points) simulation run (see *Electronic Supplementary Material*). A large

separation between the randomly picked points was used to assure 35 uncorrelated, independent points, just like in individual cardiomyocytes at the time of freezing of the myocardial tissue. The predicted distribution of the fraction of mutant *MYH7*-mRNA and β-MyHC-protein among 35 individual model-cardiomyocytes is shown in Figs. 4d and 4e. The mean fraction of mutant *MYH7*-mRNA (Figure 4d), as well as the 95% range of the 35 time points were calculated in logit space.
It should be noted that in order to account for the various experimental results (7 parameters of constraints i-v), adjustment of k_act and k_inact of the transcriptions sites and of the splicing rate k_plus_mRNA was sufficient to fit the experimental results, even though the rate constants taken from the literature were from different cell types and mostly not from cardiomyocytes. Figure S8 demonstrates that with independent, stochastic, burst-like transcription a cardiomyocyte can change, over an extended time period, from a cardiomyocyte with high expression of mutant β-MyHC to a cardiomyocyte expressing more wildtype β-MyHC and vice versa (Figure S8, bottom trace).

To estimate the expected effect of the fraction of mutant β-MyHC (protein) on the pCa_{50} we assumed (a) as a first approximation a linear decrease in pCa_{50} with increasing mutant β-MyHC-protein, (b) that the mean pCa_{50} of the controls (5.53) represents the pCa_{50} for pure wildtype cardiomyocytes (fraction of mutant protein = 0) and (c) that a pCa_{50} of 5.38, the mean value of all measured cardiomyocytes of the R723G patient, is the pCa_{50} of a mean fraction of mutant protein of 0.68, i.e., the mean fraction of mutant protein determined for the R723G patients in tissue samples (Tripathi et al., 2011). The dash-dotted line in Figure 4F is the mean pCa_{50} (5.38), the dashed lines represent the range within which 95% of all modelled data points are located (mean ± 1.96 SD). The linear relationship between pCa_{50} and the fraction of mutant β-MyHC-protein is an assumption which could not be tested experimentally. However, the results from the modelling of pCa_{50} of the individual cardiomyocytes (Figure 4F) are very similar to the data of the experiments (Figure 1C), suggesting that the relationship maybe close to linear.
Note that for comparison with experimental data (Figs. 1 and 2) we have to consider the variance in the experimental data originating from experimental error that is not included in the model-response. Total variance in pCa_{50} of cardiomyocytes from the R723G patient originates from variance due to experimental scatter (represented by the variance in pCa_{50} of control cardiomyocytes) plus additional variance from intrinsic variation among individual R723G cardiomyocytes (due to different expression of mutant β-MyHC-protein). Since both types of variance are not expected to be dependent, total variance in pCa_{50} of the R723G cardiomyocytes (SD_{tot}^2) equals intrinsic variance ($SD_{intrinsic}^2$) plus variance from experimental scatter which is equal to the variance in pCa_{50} of control cardiomyocytes ($SD_{control}^2$) with no intrinsic variance (control cardiomyocytes express no mutant β-MyHC-protein). Thus, $SD_{intrinsic}^2 = SD_{tot}^2 - SD_{control}^2$. In Figure 4F, the dotted lines include experimental scatter, i.e., represent the $mean_{experiment} \pm 1.96\ SD_{tot}$ and can be compared to experimental data in Figure 1C, upper panel.

Also, for the fraction of mutant *MYH7*-mRNA the mean and distribution predicted by the modelling (dash-dot and dashed lines in Figure 4d) is very similar to the range seen

experimentally (dash-dot and dashed lines in Figure 2c). Thus, overall, as shown in Figs. 4 and S8, stochastic, independent burst-like transcription of mutant and wildtype alleles can quite well account for all aspects of cell-to-cell variation in mutant mRNA and function, using known transcription and degradation rates of *MYH7*-mRNA as well as rates of myosin protein turnover. In other words, the modelling shows that the observed large cell-to-cell variation in mutant *MYH7*-mRNA and the large cell-to-cell functional variation among cardiomyocytes from HCM-patients can directly result from independent, stochastic, burst-like transcription of the two *MYH7* alleles, mutant and wildtype.

Statistical Analysis

Data analysis and statistics were performed using Excel, Origin, and R (R, 2013). The data are presented as mean ± SD or as mean ± 1.96 SD which represents the range within which 95% of data points are expected (see below). Mean values, SDs and p-values are given in the main text or figure legends.

Fitting of Hill equation

For each isolated cardiomyocyte, active forces generated at different Ca^{++}-concentrations (pCa-values) were normalized to the maximum force at saturating Ca^{++}-concentration (pCa 4.5). The resulting force-pCa relation follows a sigmoidal course. To characterize the sensitivity for free Ca^{++}-ions (Ca^{++}-sensitivity), the Hill function was fitted to the observed force-pCa relation and Ca^{++}-sensitivity was defined as the negative decimal logarithm of the Ca^{++} concentration at 50% force (pCa_{50} value). Fitting was performed using least-squares regression. The goodness of each fit was expressed in the corresponding coefficient of determination (R^2).

Mean values, variances, 95% prediction interval and Shapiro-Wilk test for normal distribution of normalized data

Normalized forces at partial activation (Figure 1) and the fraction of mutant mRNA of individual cardiomyocytes (Figure 2c, e) do not have normal data distributions. This is because their values are limited to the interval between 0 and 1 and our data include values near 0 and 1. We therefore applied the logit transformation as an appropriate scale transformation for statistics (Ashton, 1972) with

$$\mathrm{logit}(p) = \log(p/(1-p))$$

$$\text{and } 0 < p < 1.$$

After logit transformation all samples of normalized forces at partial activation and of the fraction of mutant mRNA showed normal data distribution according to the Shapiro-Wilk test.

The 95% prediction interval is the interval in which 95% of all measured values are expected. Presuming normal distribution, 95% of all values are within the range from mean plus 1.96 standard deviations to mean minus 1.96 standard deviations. For parameters transformed into logit space, mean values and 95% prediction interval were calculated in the logit space. For purposes of illustration and comparison lower and upper boundaries of this interval (e.g.,

dashed lines in Figure 1, Figure 2) as well as the mean (dash-dotted lines) were then back-transformed into linear space using the inverse logit transformation:

$$p = 1/(1+\exp(-\mathrm{logit}(p))).$$

t-test

After affirming normal distribution with the Shapiro-Wilk test, two-sample Student's t-tests were performed to determine whether the means of two populations were significantly different. For statistical comparison of means and variances of forces at partial activation and of pCa_{50}-values for cardiomyocytes from control-individuals vs. two R723G-patients or control cardiomyocytes vs. one A200V-patient, respectively, we put the cardiomyocytes from control-individuals in one group (Figure 1), assuming that the measurable variation between the control-individuals is solely due to experimental error. The cardiomyocytes from several controls showed a smaller variance in the measured parameters than the patient cardiomyocytes (both R723G and A200V). For the fraction of mutant mRNA, data were first logit-transformed. For $p<0.05$ significance was assumed.

F-test

Equality of variances was examined by the F-test after affirming normality with the Shapiro-Wilk test. For the fraction of mutant mRNA, the F-test was performed in logit space. For $p<0.05$ significance was assumed.

2 Supplementary Figures and Tables

2.1 Supplementary Tables

Table S1. Primers for PCR amplification.

**Primer**	**Sequence**
A200V RT	5’-GCCTTGCCTTTGCCCTTCTCA–3’
A200V F1	5'-ATCACCGGAGAATCCGGAGCA-3'
A200V R1	5'-TCCAGAAGATAGGTCTCTATGTCTG-3'
A200V F2	5’-AGGGTCATCCAGTACTTTGCTGTTATTAC-3'
A200V R2	5'-ATGTCTGCAGATGCCAACTTTCCT-3'
R723G F1	5'-GTGCTGGAGGGCATCCGCATCT-3'
R723G R1	5'-CTTTTTGTACTCCATTCTGGCGAGCACA-3
R723G F2	5'-CCAACCGCATCCTCTACGGGGACTTCCGGCAGAGGGAT-3'’
R723G R2	5'-CCATTCTGGCGAGCACACCT-3'
MYH7-Real-Time RT	5’–CTTTGCCCTTCTCAATAGGCGCATCAG–3’
MYH7-Real-Time F	5’–AACATCATTGGCTGGCTGCAGAA–3’
MYH7-Real-Time R	5’–CATAGTTGGCAAACAGGGTGCT–3’
MYH7-Real-Time probe	5’–6-FAM-TCAATGAGACTGTCGTGGGCTTGTATCAGAAGT-TAMRA–3’

Table S2. Mutant and wildtype peptides for β-MyHC-protein quantification in myocardial tissue with mutation A200V.

Table shows sequence and molecular mass of native wildtype and mutant peptides after trypsin-digest, as well as the *m/z* ratio of the 2-fold positively charged species of both wildtype and stable-isotope-labeled internal standard peptides. The amino acid exchange of alanine vs. valine is indicated by bold letters.

	**Sequence and target peptide ions $[M + 2H^+]$ for quantification**	**Deconvoluted monoisotopic mass [Da] of native peptides**
Wildtype peptide	VIQYF*AVIA**A**IGDR<br>native ion: *m/z* 768.43<br>isotope-labeled ion: *m/z* 773.43	1534.86
Mutant peptide	VIQYF*AVIA**V**IGDR<br>native ion: *m/z* 782.44<br>isotope-labeled ion: *m/z* 787.44	1562.89

* indicates the phenylalanine (F) which in the internal standard peptides was replaced by an isotopically enriched phenylalanine (^{13}C, ^{15}N).

Table S3. Rate constants used in the model calculations.

activation of transcription (k_act)	0.018 /h[#]
inactivation of transcription (k_inact)	0.72 /h[#]
synthesis of mutant pre-mRNA (k_plus_pre-mRNA_mut)	1800 mRNA/h[†]
synthesis of wildtype pre-mRNA (k_plus_pre-mRNA_wt)	1800 mRNA/h[†]
generation of mutant mRNA (k_plus_mRNA_mut)	0.3 /h[#]
generation of wildtype mRNA (k_plus_mRNA_wt)	0.3 /h[#]
degradation of mutant mRNA (k_minus_mRNA_mut)	0.06 /h[‡]
degradation of wildtype mRNA (k_minus_mRNA_wt)	0.12 /h[‡]
synthesis of mutant protein (k_plus_Protein_mut)	2400 protein/mRNA /h[§]
synthesis of wildtype protein (k_plus_Protein_wt)	2400 protein/mRNA /h[§]
degradation of mutant protein (k_minus_Protein_mut)	0.012 /h[‖]
degradation of wildtype protein (k_minus_Protein_wt)	0.012 /h[‖]

[#] Best fit from modelling

[†] k_plus_pre_mRNA = mRNA molecules per cell (median=589; Figure 3) * k_minus_mRNA (mean=0.064/h) * k_inact/k_act (40)

[‡] (Sharova et al., 2009; Schwanhausser et al., 2011)

[§] (Everett et al., 1983)

[‖] (Schwanhausser et al., 2011)

2.2 Supplemental Figures

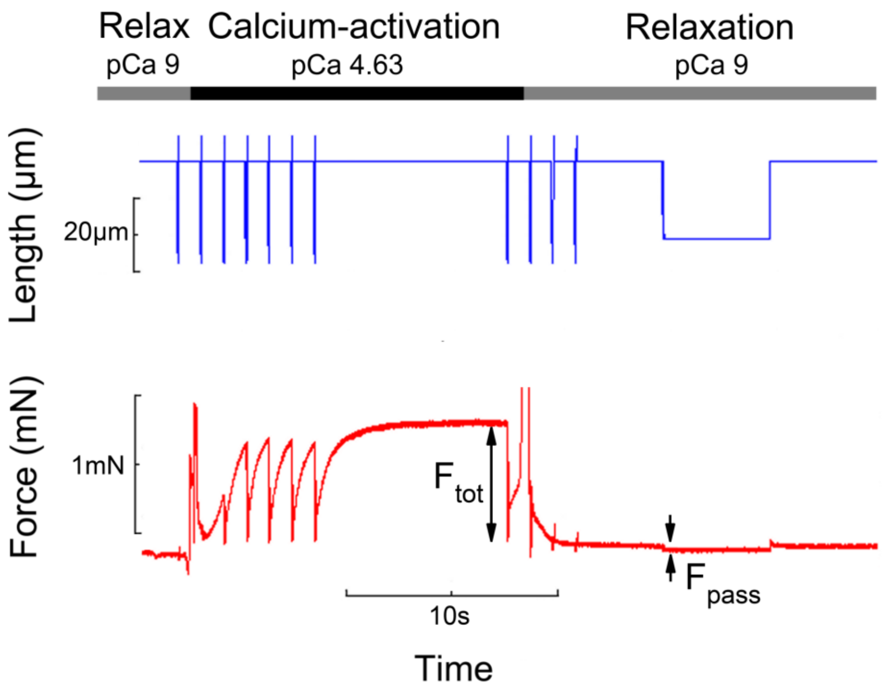

Figure S1

Force measurements of single, chemically permeabilized cardiomyocytes.

Original length and force record showing the experimental protocol as it was used for single cardiomyocytes with mutation A200V. After moving the cardiomyocyte from relaxing solution to Ca^{++}-activation, initially several quick length releases of the cardiomyocyte were applied where force briefly drops to zero. This stabilizes the striation pattern and allows complete Ca^{++}-equilibration before full force development is allowed to reach the plateau. To determine F_{tot}, another quick release was applied once steady state isometric force had been reached. Subsequently the cardiomyocyte was moved back into relaxing solution. Once the cardiomyocyte had fully relaxed, a step release and re-stretch was applied to record passive force (F_{pass}). The small upward shift of the zero force level when the cardiomyocyte is moved from relaxing solution to the trough with activating solution is due to changes in surface tension of the solution in the different troughs. The zero force level is indicated by the force level during the releases when the cardiomyocyte is unloaded.

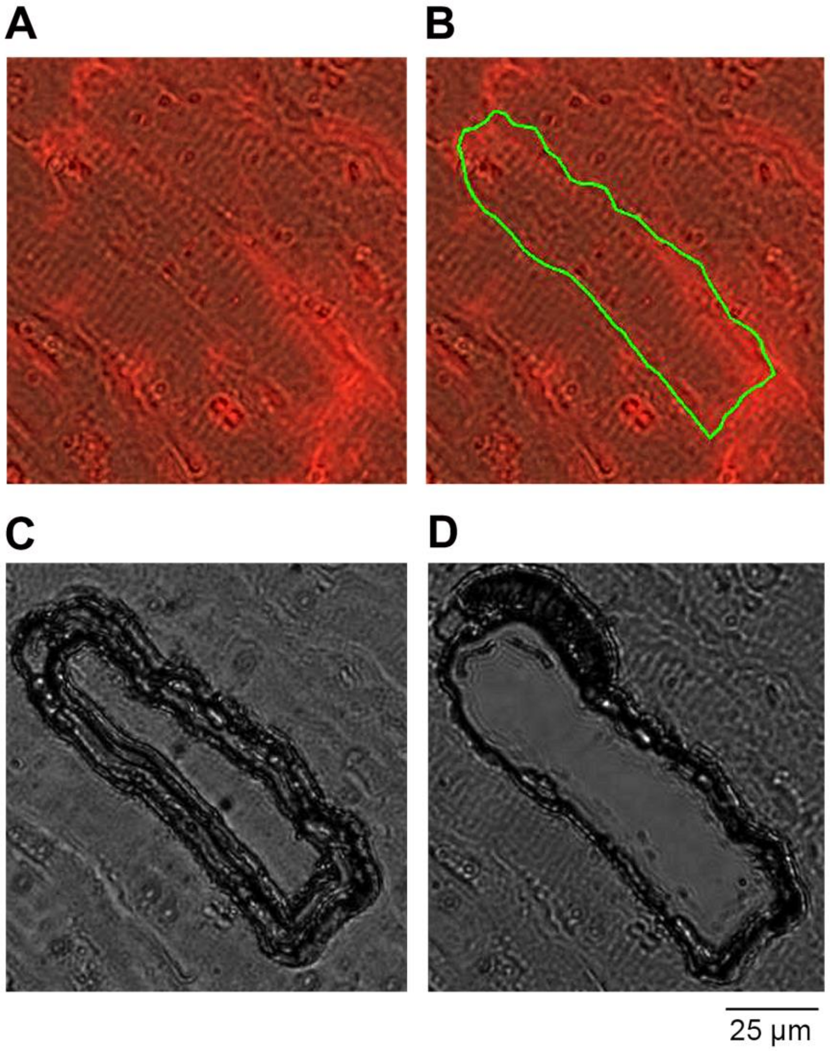

Figure S2

Isolation of individual cardiomyocytes from cryosections by laser capture microdissection.

(A) Superimposed fluorescence and bright field images of a cryosection of left ventricular myocardium. Intercalated discs marked with anti-cadherin antibody visualized by a TRITC-labelled secondary antibody. Cell borders of a cardiomyocyte delineated by superposition of fluorescence signal and striation pattern. **(B)** Individual cardiomyocyte marked for dissection by UV laser, **(C)** dissected cell segment, **(D)** void area after cell segment was catapulted into nuclease free water in the lid of a PCR tube by a pulse of defocused UV-light.

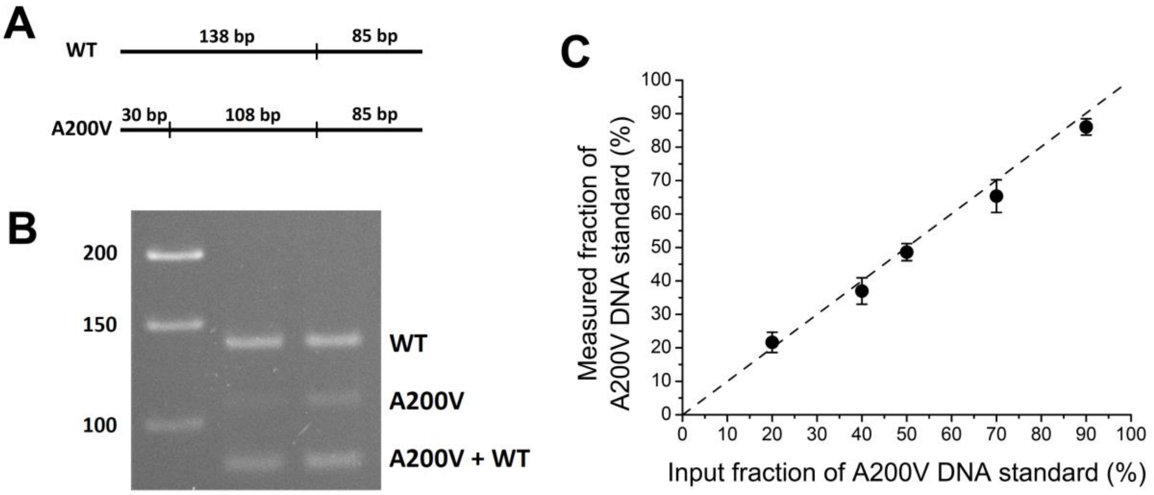

Figure S3

Control of linearity of restriction digest approach for MYH7-mRNA-quantification of mutation A200V.

(A) *Schematic* of *Hpy*4CHI-restriction sites of PCR-product with heterozygous β-MyHC-mutation A200V: 85bp fragment for both alleles, 108bp fragment specific for mutant, and 138bp fragment specific for wildtype. **(B)** Agarose gel of restriction digests used to test linearity of the quantification procedure for mutation A200V, as shown for other mutations (Tripathi et al., 2011). *Left lane*, equimolar standard ladder; *middle and right lane*, 80:20 and 60:40 mixtures of wildtype and mutant synthetic plasmids. **(C)** Analysis of whole set of defined mixtures of mutant and wildtype synthetic plasmids. *Dashed line*, expected data if input and measured values were identical. *Filled circles*, experimentally determined percentage of mutated templates plotted vs. percentage of mutated templates put into mixtures from at least four independent experiments (measured fraction of mutated DNA (%) ± SD).

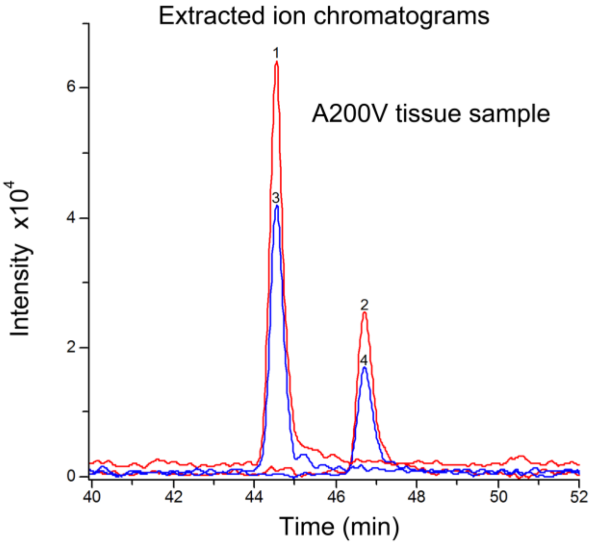

Figure S4

Quantification of wildtype and A200V-mutated β-MyHC-peptides by mass spectrometry.

Myosin extracted from cardiac samples was spiked with equimolar amounts of stable-isotope labelled internal standard peptides and digested with trypsin before analysis by nanoLC-ESI-MS. *Red trace*, extracted ion chromatograms (EICs) of isotope-labelled synthetic peptides. Peak 1 (m/z = 773.43) wildtype peptide; peak 2 (m/z = 787.44) mutant peptide. *Blue trace*, EICs of native peptides; peak 3 (m/z = 768.43) wildtype peptide; peak 4 (m/z = 782.44) mutant peptide. Note that integrated peak areas of the isotope labelled synthetic standard peptides, although added in equimolar amounts, are quite different between mutant and wildtype peptides. This may be due to, e.g., different competition for ionization at the time when mutant and wildtype peptides eluate from the LC-column, or differential adsorption of mutant and wildtype peptides during preparation even in low absorption vials. Since native and isotope labelled forms eluate at the same time and the isotope labelled peptides were present already during the digest, the ratio of peak areas of the equimolar isotope labelled peptides allows accounting for any differences in behavior between mutant and wildtype peptides throughout the quantification procedure. To correct for such effects on the native peptides, the ratio of peak areas of mutant/wildtype native peptides was normalized to the intensity ratio observed for the isotope labelled synthetic standard peptides.

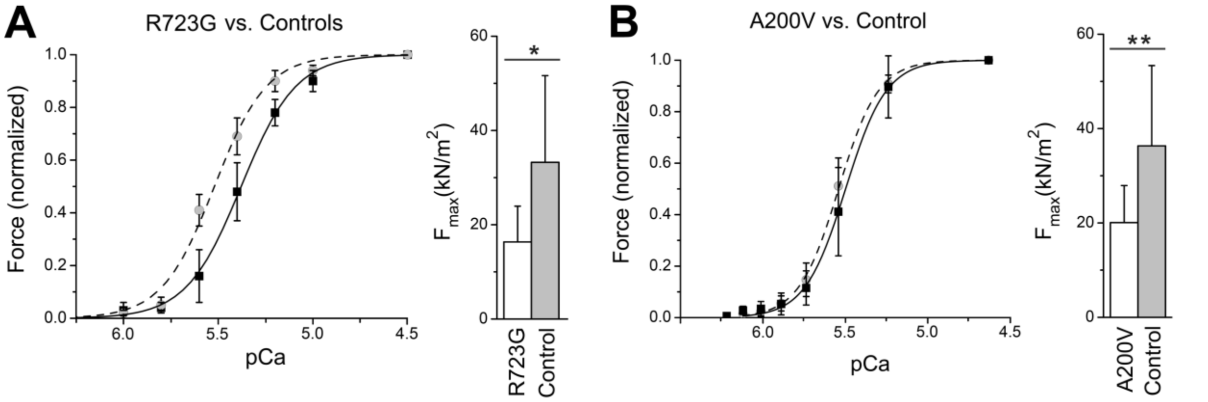

Figure S5

Mean force-pCa relations and absolute forces generated by cardiomyocytes with β-MyHC-mutations R723G and A200V.

(**A**) Force-pCa relations of cardiomyocytes with myosin mutation R723G (22 cardiomyocytes; filled black symbols, solid line) vs. controls (8 cardiomyocytes; filled gray symbols, dashed solid line). The mutation causes a statistically significant shift of the force-pCa relation to the right, i.e., to significantly lower pCa_{50}-values ($p = 0.03$; paired t-test (Galbraith et al., 2010)). This indicates, on average, a reduced Ca^{++}-sensitivity compared to controls. *Right panel*, absolute forces generated at saturating Ca^{++}-concentrations (means ± SD). R723G myocytes generate on average significantly lower forces compared to controls ($p = 0.018$; t-test) **(B)** Mean force-pCa relations of 19 cardiomyocytes with myosin mutation A200V (black filled symbols, solid line) vs. 17 control cardiomyocytes (grey filled symbols, dashed line). On average, the mean force pCa-relation for A200V-myocytes is shifted to the right. *Right panel*, absolute forces at saturating Ca^{++}-concentrations which are only about half the forces seen for controls ($p = 0.0016$; t-test). Note, that for both R723G and A200V cardiomyocytes the standard deviations of normalized forces at the different Ca^{++}-concentrations are larger than for control cardiomyocytes. This is particularly prominent and statistically significant (F-test) for A200V cardiomyocytes, and results from the larger cell-to-cell variation in the R723G and A200V cardiomyocytes, respectively (cf. Figure 1a, b). As a consequence of the larger cell-to-cell variability, the average shift of the force-pCa relation of the A200V cardiomyocytes to the right, i.e., to lower Ca^{++}-sensitivity is smaller and statistically not significant.

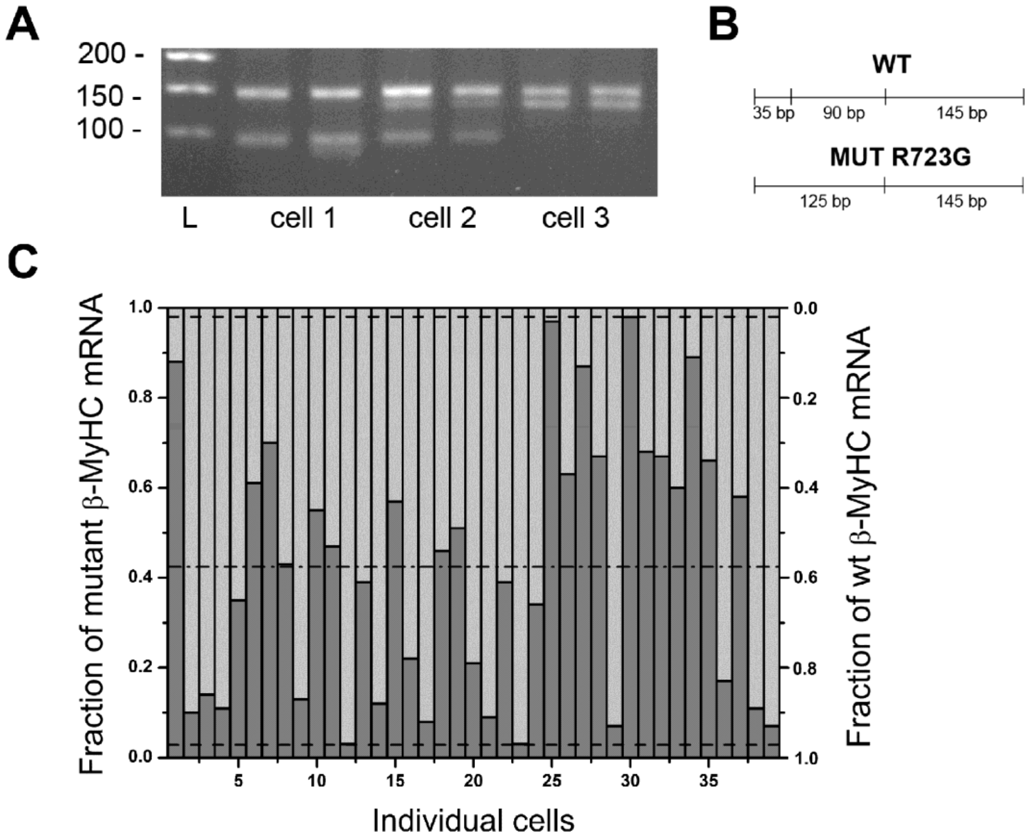

Figure S6

Large variability in fraction of R723G-mRNA and wildtype MYH7-mRNA in individual cardiomyocytes microdissected from cryosections of myocardium.

(A) Sample gel of restriction digest products of 3 individual cardiomyocytes microdissected from cryo-sections of R723G-myocardium (patient III-1, family 157 in (Enjuto et al., 2000)). Lysate of each cell was divided in 2 equal aliquots and analyzed in parallel; L, DNA standard ladder. Note the marked differences in band pattern among the individual cardiomyocytes (cell 1-3) while band patterns of the two aliquots of each cell are rather similar. **(B)** Schematic of NheII-restriction sites which yields: 90bp band, from wildtype *MYH7*-mRNA; 125 bp band, from mutant *MYH7*-mRNA; 145bp band, from mutant and wildtype mRNA; 35 bp band, from wildtype *MYH7*-mRNA but outside range of gels. **(C)** Fraction of R723G-mRNA (dark gray bars; y-axis on the left of panel) and of wildtype-mRNA (light gray bars; y-axis on the right of panel) in 39 cardiomyocytes from left ventricular free wall of R723G-myocardium. *MYH7*-mRNA varies from almost pure wildtype to pure mutant.

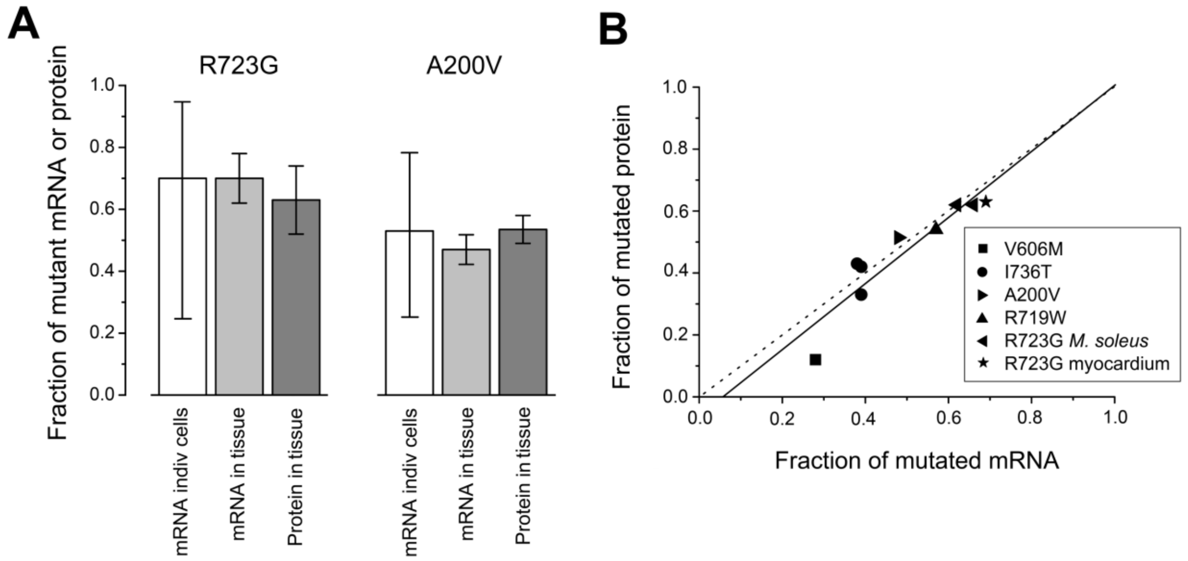

Figure S7

Microdissected cardiomyocytes represent fractions of mutated MYH7-mRNA of all cardiomyocytes within a tissue sample; fractions of mutated β-MyHC-protein and of mutated MYH7-mRNA are very similar for several missense mutations.

(A) Quantification of the fraction of mutated *MYH7*-mRNA and mutated β-MyHC (protein) for mutation R723G and A200V. *White columns*, average of mutated mRNA in single cardiomyocytes (for R723G the average fraction is 0.70 +SD 0.25 –SD 0.45 (±SD here from logit-calculation in Figure 2c), n=35 cardiomyocytes; for A200V the average fraction is 0.53 +SD 0.25 –SD 0.28 (±SD here from logit-calculation in Figure 2e), n=21 cardiomyocytes; *light gray columns*, mutated mRNA in whole tissue sections (for R723G the fraction is 0.70±0.08, n=5 sections; for A200V the fraction is 0.47±0.05, n=3 sections); *dark gray columns*, fraction of mutated β-myosin protein in tissue samples as determined by mass spectrometry. For R723G the fraction is 0.63±0.11, n=2 peptide samples (digests), data from (Tripathi et al., 2011); for A200V the fraction is 0.54±0.045, n=5 peptide samples. **(B)** Summary of the fraction of mutant β-MyHC-protein vs. fraction mutant *MYH7*-mRNA in tissue samples of different HCM-related mutations in the head domain of the β-MyHC (all data except for mutation A200V are from (Tripathi et al., 2011)). Filled symbols, mean values of tissue samples or whole cryosections; solid line, linear fit to the data points; dashed line represents exact 1:1 relation between fraction of mutated mRNA and fraction of mutated protein.

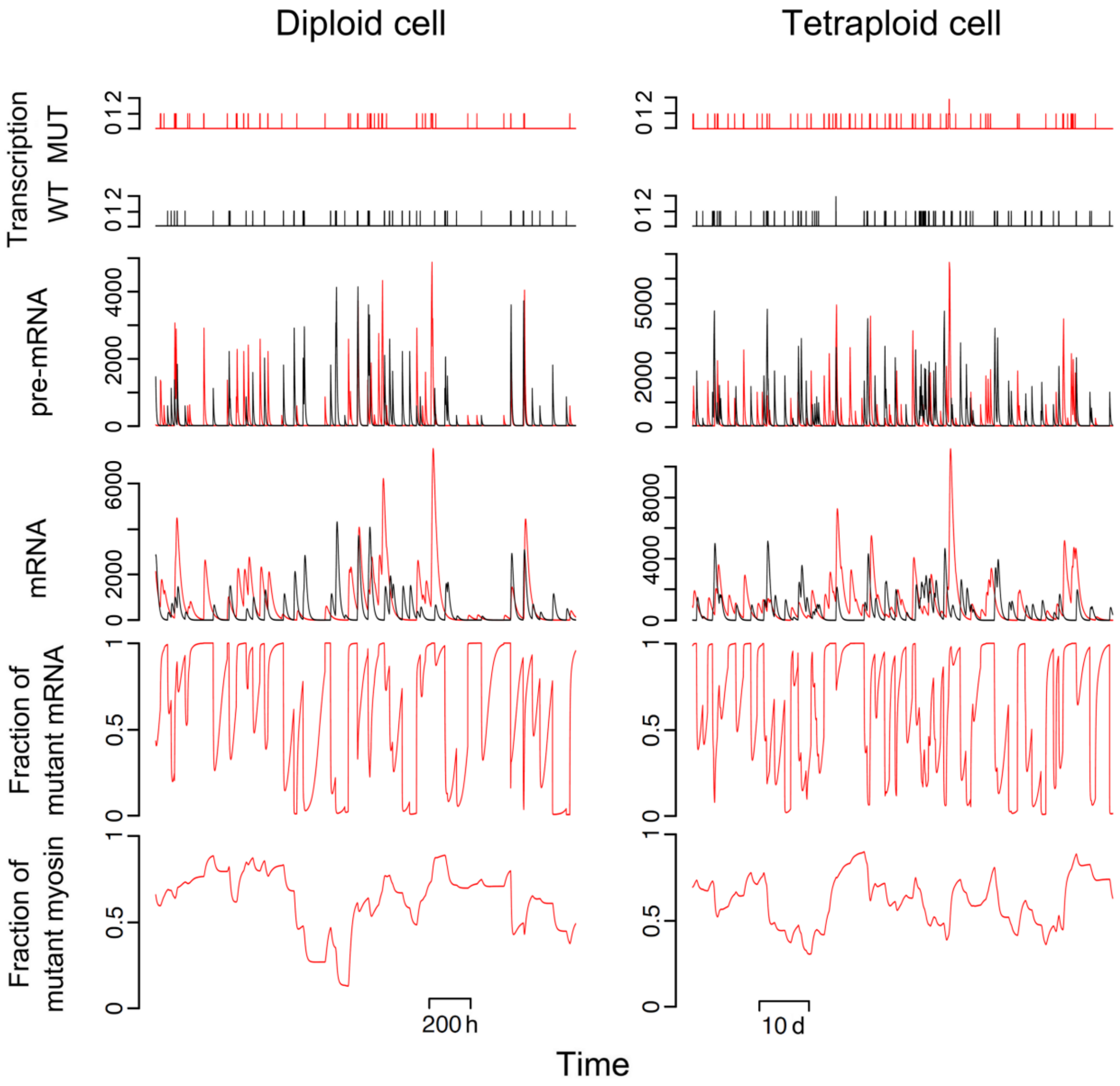

Figure S8

Time-periods of 80 days of model output for a diploid (left) and a tetraploid (right) cardiomyocyte, each heterozygous for β-MyHC-mutation R723G.

Two examples of time courses predicted by stochastic, burst like transcription of *MYH7* which is independent for the mutant (MUT) and the wildtype (WT) allele in heterozygous cardiomyocytes. Panels from top to bottom show: random on/off switching of allele transcription (note that for the tetraploid cell there are two mutant and two wildtype alleles. Hence, 0, 1, and 2 represent the number of switched on alleles), resulting time course of the number of mutant and wildtype pre-mRNA molecules and mRNA molecules, fraction of mutant mRNA, and fraction of mutant myosin molecules over a time period of 80 days. Higher ploidy results in more frequent transcription pulses and somewhat smoother time courses for fraction of mutant mRNA and myosin.

3 Supplemental References

Ashton, W.D. (1972). *The logit transformation with special reference to its uses in bioassay (Griffin's statistical monographs & courses, no. 32).* London: Hafner Pub. Co.

Bahar Halpern, K., Tanami, S., Landen, S., Chapal, M., Szlak, L., Hutzler, A., et al. (2015). Bursty gene expression in the intact mammalian liver. *Mol Cell* 58(1)**,** 147-156. doi: 10.1016/j.molcel.2015.01.027.

Becker, E., Navarro-Lopez, F., Francino, A., Brenner, B., and Kraft, T. (2007). Quantification of mutant versus wild-type myosin in human muscle biopsies using nano-LC/ESI-MS. *Anal Chem* 79(24)**,** 9531-9538. doi: 10.1021/ac701711h.

Belin, R.J., Sumandea, M.P., Kobayashi, T., Walker, L.A., Rundell, V.L., Urboniene, D., et al. (2006). Left ventricular myofilament dysfunction in rat experimental hypertrophy and congestive heart failure. *Am J Physiol Heart Circ Physiol* 291(5)**,** H2344-2353. doi: 10.1152/ajpheart.00541.2006.

Bergmann, O., Zdunek, S., Alkass, K., Druid, H., Bernard, S., and Frisen, J. (2011). Identification of cardiomyocyte nuclei and assessment of ploidy for the analysis of cell turnover. *Exp Cell Res* 317(2)**,** 188-194. doi: 10.1016/j.yexcr.2010.08.017.

Boon, W.C., Petkovic-Duran, K., White, K., Tucker, E., Albiston, A., Manasseh, R., et al. (2011). Acoustic microstreaming increases the efficiency of reverse transcription reactions comprising single-cell quantities of RNA. *Biotechniques* 50(2)**,** 116-119. doi: 10.2144/000113587.

Brenner, B. (1983). Technique for stabilizing the striation pattern in maximally calcium-activated skinned rabbit psoas fibers. *Biophys J* 41(1)**,** 99-102. doi: 10.1016/S0006-3495(83)84411-7.

Brodsky, V., Sarkisov, D.S., Arefyeva, A.M., Panova, N.W., and Gvasava, I.G. (1994). Polyploidy in cardiac myocytes of normal and hypertrophic human hearts; range of values. *Vichows Archiv A Pathol Anat* 424(4)**,** 429-435. doi:10.1007/BF00190566.

Enjuto, M., Francino, A., Navarro-Lopez, F., Viles, D., Pare, J.C., and Ballesta, A.M. (2000). Malignant hypertrophic cardiomyopathy caused by the Arg723Gly mutation in beta-myosin heavy chain gene. *J Mol Cell Cardiol* 32(12)**,** 2307-2313. doi: 10.1006/jmcc.2000.1260.

Everett, A.W., Clark, W.A., Chizzonite, R.A., and Zak, R. (1983). Change in synthesis rates of alpha- and beta-myosin heavy chains in rabbit heart after treatment with thyroid hormone. *J Biol Chem* 258(4)**,** 2421-2425. PMID: 6337155.

Fohr, K.J., Warchol, W., and Gratzl, M. (1993). Calculation and control of free divalent cations in solutions used for membrane fusion studies. *Methods Enzymol.* 221**,** 149-157.

Galbraith, S., Daniel, J.A., and Vissel, B. (2010). A study of clustered data and approaches to its analysis. *J Neurosci* 30(32)**,** 10601-10608. doi: 10.1523/JNEUROSCI.0362-10.2010.

Hamdani, N., Borbely, A., Veenstra, S.P., Kooij, V., Vrydag, W., Zaremba, R., et al. (2010). More severe cellular phenotype in human idiopathic dilated cardiomyopathy compared to ischemic heart disease. *J Muscle Res Cell Motil* 31(4)**,** 289-301. doi: 10.1007/s10974-010-9231-8.

Herget, G.W., Neuburger, M., Plagwitz, R., and Adler, C.P. (1997). DNA content, ploidy level and number of nuclei in the human heart after myocardial infarction. *Cardiovasc Res* 36(1)**,** 45-51. doi: 10.1016/S0008-6363(97)00140-5.

Kraft, T., Montag, J., Radocaj, A., and Brenner, B. (2016). Hypertrophic Cardiomyopathy: Cell-to-Cell Imbalance in Gene Expression and Contraction Force as Trigger for

Disease Phenotype Development. *Circ Res* 119(9), 992-995. doi: 10.1161/CIRCRESAHA.116.309804.

Kraft, T., Paalberends, E.R., Boontje, N., S., T., Brandis, A., Montag, J., et al. (2013). Familial Hypertrophic Cardiomyopathy: Functional effects of myosin mutation R723G in cardiomyocytes. *J Mol Cell Cardiol* 57, 13-22. doi: 10.1016/j.yjmcc.2013.01.001.

Lyubimova, A., Itzkovitz, S., Junker, J.P., Fan, Z.P., Wu, X., and van Oudenaarden, A. (2013). Single-molecule mRNA detection and counting in mammalian tissue. *Nat Protoc* 8(9), 1743-1758. doi: 10.1038/nprot.2013.109.

R, T.C. (2013). "R Core Team. A language and environment for statistical computing", (ed.) R. R Foundation for Statistical Computing, Vienna, Austria. http://www.R-project.org/.).

Raj, A., Peskin, C.S., Tranchina, D., Vargas, D.Y., and Tyagi, S. (2006). Stochastic mRNA synthesis in mammalian cells. *PLoS Biol* 4(10), e309. doi: 10.1371/journal.pbio.0040309.

Schwanhausser, B., Busse, D., Li, N., Dittmar, G., Schuchhardt, J., Wolf, J., et al. (2011). Global quantification of mammalian gene expression control. *Nature* 473(7347), 337-342. doi: 10.1038/nature10098.

Sharova, L.V., Sharov, A.A., Nedorezov, T., Piao, Y., Shaik, N., and Ko, M.S. (2009). Database for mRNA half-life of 19 977 genes obtained by DNA microarray analysis of pluripotent and differentiating mouse embryonic stem cells. *DNA Res* 16(1), 45-58. doi: 10.1093/dnares/dsn030.

Thompson, J.R., Marcelino, L.A., and Polz, M.F. (2002). Heteroduplexes in mixed-template amplifications: formation, consequence and elimination by 'reconditioning PCR'. *Nucleic Acids Res* 30(9), 2083-2088. PMCID: PMC113844.

Tripathi, S., Schultz, I., Becker, E., Montag, J., Borchert, B., Francino, A., et al. (2011). Unequal allelic expression of wild-type and mutated beta-myosin in familial hypertrophic cardiomyopathy. *Basic Res Cardiol* 106, 1041-1055. doi:10.1007/s00395-011-0205-9.

van der Velden, J., Papp, Z., Zaremba, R., Boontje, N.M., de Jong, J.W., Owen, V.J., et al. (2003). Increased Ca2+-sensitivity of the contractile apparatus in end-stage human heart failure results from altered phosphorylation of contractile proteins. *Cardiovasc Res* 57(1), 37-47. doi: 10.1016/S0008-6363(02)00606-5.

WMA (1997). World Medical Association declaration of Helsinki. *Cardiov. Res.* 35, 2-3. doi: 10.1016/S0008-6363(97)00109-0.
